# Replication-competent SIVcpz CRISPR screen identifies barriers to successful cross-species transmission

**DOI:** 10.1128/jvi.00314-26

**Published:** 2026-05-07

**Authors:** Qinya Xie, Qingxing Wang, Sabrina Noettger, Guillermo Gosálbez, Annika C. Betzler, Meta Volcic, Dorota Kmiec, Stefan Krebs, Alexander Graf, Dila Gülensoy, Gilbert Weidinger, Konstantin M. J. Sparrer, Frank Kirchhoff

**Affiliations:** 1 Institute of Molecular Virology, Ulm University Medical Center 27197, Ulm, Germany; 2 Laboratory for Functional Genome Analysis, Gene Center, LMU Munich 9183 , Munich, Germany; 3 Institute of Biochemistry and Molecular Biology, Ulm University 9189, Ulm, Germany; 4 German Center for Neurodegenerative Diseases (DZNE), Ulm, Germany; The Ohio State University, Columbus, Ohio, USA

**Keywords:** SIVcpz, replication-competent CRISPR screen, IFITM2, PCED1B, MEFV, AXIN1

## Abstract

Simian immunodeficiency viruses (SIVs) have crossed from apes to humans at least four times, but only one event gave rise to the AIDS pandemic. The host barriers that pandemic HIV-1 group M (*major*) strains overcame to spread efficiently in humans remain poorly understood. To identify such barriers, we performed CRISPR-Cas9 screens driven by the replication efficiency of SIVcpz, the chimpanzee precursor of HIV-1. Guide RNA libraries targeting more than 500 human genes encoding potential antiviral factors were inserted into the replication-competent SIVcpz MB897 molecular clone, which is phylogenetically closely related to HIV-1 group M strains. Propagation in Cas9-expressing human SupT1 T cells significantly enriched for sgRNAs targeting *AXIN1, CEACAM3, CD72, EHMT2, GRN, HMOX1, HMGA1, ICAM2, IFITM2, MEFV, PCED1B, SGOL2, SMARCA4, SUMO1,* and *TMEM173*. These hits only partially overlapped with those identified in analogous HIV-1-based screens, indicating virus-specific restriction profiles. Functional analyses confirmed that IFITM2 (interferon-induced transmembrane protein 2), PCED1B (PC-esterase domain-containing protein 1B), MEFV (Mediterranean fever protein, pyrin/TRIM20), and AXIN1 (Axis inhibition protein 1) restrict replication of the analyzed SIVcpz strains but not HIV-1 group M strains in primary human CD4^+^ T cells. These findings reveal previously unrecognized host factors that limit SIVcpz replication in human cells and highlight barriers that at least some HIV-1 group M strains overcame during adaptation for pandemic spread.

**IMPORTANCE:**

Four independent transmission events of simian immunodeficiency viruses from chimpanzees and gorillas to humans gave rise to human immunodeficiency virus type 1, but only one led to the global AIDS pandemic. Understanding which adaptations allowed the pandemic HIV-1 M strains to spread efficiently in humans remains a key question in virus evolution and public health. In this study, we engineered replication-competent SIVcpz constructs carrying more than 1,500 single-guide RNAs to identify antiviral genes in Cas9-expressing cells. This approach revealed several cellular factors that restrict SIVcpz but not the pandemic HIV-1 M strains analyzed in primary human T cells. These findings provide new insights into antiviral defense mechanisms and the adaptations that most likely contributed to the efficient spread of HIV-1.

## INTRODUCTION

Viral pathogens and their hosts are engaged in a continuous evolutionary arms race (1). Functionally and structurally diverse host-encoded antiviral factors provide rapid, broad-spectrum protection against infection (2–4). In response, viruses evolved strategies to evade or counteract these defenses, leading to a dynamic interplay that shapes infection outcomes (3, 5, 6). Failures in antiviral host defense can result in severe disease and—in very rare cases—global pandemics (7, 8).

HIV-1, the causative agent of AIDS, emerged through four cross-species transmissions of simian immunodeficiency viruses infecting chimpanzees (SIVcpz) and western lowland gorillas (SIVgor) (8, 9). However, only one transmission of SIVcpz to humans, originating from a central chimpanzee (*Pan troglodytes troglodytes, Ptt*) about 100 years ago that gave rise to the M (*major*) group of HIV-1, is responsible for the AIDS pandemic (9). In contrast, SIVcpz strains from eastern chimpanzees (*Pan troglodytes schweinfurthii, Pts*) have not been detected in humans (10), indicating the importance of both viral and host-specific factors for successful zoonotic transmission. One reason for the efficient spread of HIV-1 group M strains is their ability to counteract human restriction factors. For example, their accessory protein Vpu evolved to antagonize tetherin (BST-2), a host factor that inhibits virion release (11, 12). Because HIV-1 group M strains are already well adapted to the human host and effectively evade innate immune defenses, they are poorly suited for identifying the antiviral mechanisms that were overcome during their emergence.

To identify antiviral defenses that posed early barriers to efficient spread of HIV-1 in humans, we adapted our previously developed CRISPR-based screening approach (13) by replacing pandemic HIV-1 group M constructs with a closely related SIVcpz*Ptt* strain. Unlike HIV-1 group M strains, SIVcpz is not adapted to human cells and, thus, provides a useful tool for identifying antiviral mechanisms that were overcome during HIV-1 adaptation. These factors may continue to limit lentiviral zoonoses and restrict replication of less-adapted HIV-1 lineages, including group O, N, and P viruses. We employed the ‘traitor virus’ (TV) approach, in which replication-competent infectious molecular clones (IMCs) are engineered to express single guide RNAs (sgRNAs) targeting host genes. In Cas9-expressing T cells, viruses encoding sgRNAs against antiviral genes gain a replication advantage, enabling identification of restriction factors based on sgRNA enrichment during serial viral passaging (13). Unlike conventional overexpression, RNAi, or single-cycle CRISPR screens, the TV strategy does not require manipulation of cellular factors prior to infection of the cells and is driven by viral replication efficacy. This allows discovery of host genes with modest effects in single-round assays but substantial impact across multiple replication cycles. Unlike most previous screens that focused on early-acting restriction factors, the TV approach uncovers antiviral mechanisms acting after proviral integration, such as viral transcription, translation, processing, trafficking, assembly, release, and virion infectivity. Thus, it covers large parts of the viral replication cycle and more closely reflects the dynamics of viral spread *in vivo*.

We previously used libraries of replication-competent HIV-1 constructs encoding >1,500 sgRNAs for virus-driven discovery of antiviral factors (13). Here, we extended this technique to SIVcpz*Ptt* to uncover antiviral factors and mechanism that pandemic HIV-1 strains evolved to counteract or evade during adaptation to the human host. Propagation of SIVcpz*Ptt* in Cas9-expressing SupT1-R5 cells led to the enrichment of sgRNAs targeting host factors that overlapped with but were distinct from those previously identified in HIV-1-based screens. Functional analyses confirmed that several of these factors restrict SIVcpz*Ptt* but not HIV-1 in primary human CD4+ T cells. Our results reveal barriers to successful lentiviral zoonoses and provide insights into the host determinants that shaped the emergence and pandemic potential of HIV-1.

## RESULTS

### SIVcpz*Ptt* MB897 replicates efficiently in SupT1-R5 Cas9 cells

**Fig 1:**
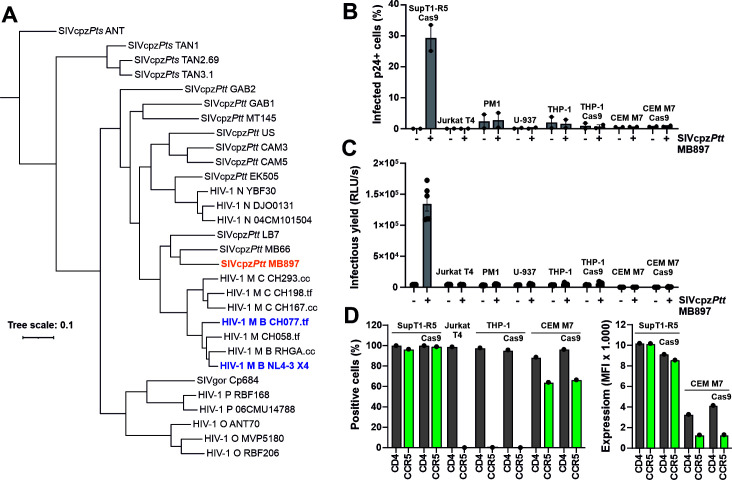
Phylogenetic relationship and infectivity of SIVcpz*Ptt* MB897. (**A**) Evolutionary relationship of SIVcpz and SIVgor strains with M, N, O, and P strains of HIV-1. The tree was constructed based on the genomic sequences using the maximum likelihood method (17). Scale bar: 0.1 nucleotide replacements per site. The SIVcpzPtt MB897, HIV-1 CH077, and NL4-3 IMCs used in the present and previous screens are highlighted in orange and blue, respectively. Tf, transmitted-founder; cc, chronic control. (**B**) Percentages of cells productively infected with SIVcpz*Ptt* MB897 (p24+ and CD4 low). Infection was determined by flow cytometric analysis at three days post-infection (Fig. S1). (**C**) Infectious SIVcpz*Ptt* MB897 production was determined by infecting TZM-bl reporter cells with supernatants obtained from the indicated cell lines 5 days after virus exposure. (**D**) CD4 and CCR5 expression in the indicated cell lines was determined by flow cytometry. Shown are the percentages of CD4- and CCR5-positive cells (left) and the mean fluorescence intensities for SupT1-R5 and CEM-M7 cells (right).

To uncover host factors that restricted early spread of HIV-1, we performed a CRISPR-based screen using the SIVcpz*Ptt* MB897 infectious molecular clone (IMC). This SIVcpz strain was selected because it is genetically closely related to pandemic HIV-1 M strains, including the NL4-3 and CH077 IMCs used in our initial screen (Fig. 1A), and replicates in human cells as well as in humanized mice (14–16).

To identify suitable human cell lines for virus propagation, we generated SIVcpz*Ptt* MB897 stocks by transfection of HEK 293T cells and used them to infect SupT1-R5, Jurkat, PM1, U-937, THP-1, and CEM-M7 cells. SupT1-R5 (modified to express CCR5) and Jurkat cells were derived from T-cell leukemias (16, 18), and PM1 cells from peripheral blood lymphocytes (19). CEM-M7 is a CEM subclone with enhanced susceptibility to interferon (IFN) (20). U-937 and THP-1 are monocytic leukemia lines that can differentiate into macrophage-like cells. We previously generated Cas9-expressing derivatives of SupT1-R5 and CEM-M7 cells (13).

Productive infection was assessed by flow-cytometric analysis of intracellular p24 capsid antigen expression and CD4 down-modulation by Nef (Fig. S1). We found that only SupT1-R5 cells were susceptible to SIVcpz*Ptt* MB897 infection (Fig. 1B). To further validate this, supernatants from all cell cultures were used to infect TZM-bl cells, which express CD4, CCR5, as well as CXCR4, and contain Tat-responsive β-galactosidase and luciferase reporters (21). Supernatants from SupT1-R5 Cas9 cells induced high β-galactosidase activity (Fig. 1C), indicating effective production of infectious SIVcpz*Ptt* MB897. To determine the basis for the differential susceptibility of these cell lines to infection, we performed FACS analyses of CD4 and CCR5, the major coreceptor of SIVcpz (17). The results showed that only SupT1-R5 and CEM-M7 cells express CCR5 (Fig. 1D). Consistent with their differing susceptibilities to SIVcpz infection, CCR5 expression levels were substantially higher in SupT1-R5 than in CEM-M7 cells (Fig. 1D, right panel). Therefore, SupT1-R5 Cas9 cells were used in subsequent experiments.

### Generation of sgRNA-expressing SIVcpz*Ptt* MB897 constructs

**Fig 2:**
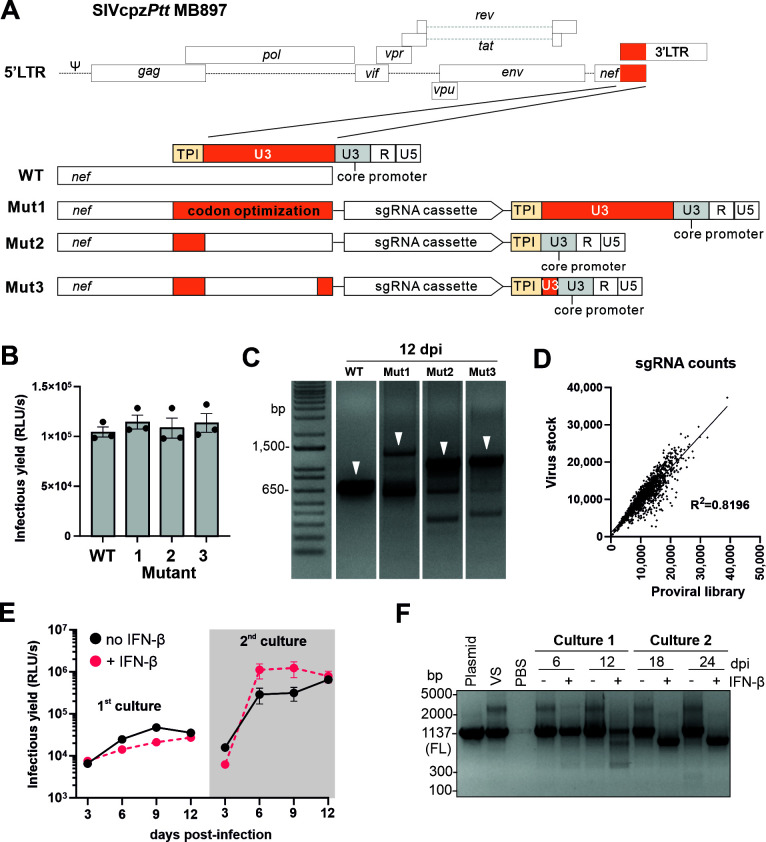
Generation and characterization of SIVcpz*Ptt* MB897 sgRNA constructs. (**A**) Schematic of sgRNA expressing SIVcpz*Ptt* MB897 IMCs. The sgRNA expression cassette was inserted between *nef* and the 3′LTR. Mut1 contains a duplicated nef/3'LTR overlap with codon-optimized *nef*. Mut2 and Mut3 lack parts of U3 that serve mainly as Nef coding region but maintain critical *cis* regulatory element: a T-rich region, the polypurine tract, and attachment sites required for integration, named the TPI region. (**B**) Infectious virus yield from HEK293T cells transfected with parental or Mut1–Mut3 constructs, measured by TZM-bl reporter assay (mean ± SD of three experiments). (**C**) Stability of the constructs in SupT1-CCR5-Cas9 cells, assessed by RT-PCR 12 dpi (days post-infection). Arrows indicate expected fragment sizes. (**D**) Correlation between sgRNA read counts in viral stocks and proviral DNA from the MB897 sgRNA library. (**E**) Infectious virus production in SupT1-R5 Cas9 cells infected with the SIVcpz*Ptt* MB897mut3-sgRNA library, passaged as indicated in Fig. S2. Virus titers in supernatants were measured by TZM-bl assay. (**F**) RT-PCR analysis of MB897mut3-sgRNA constructs in the corresponding supernatants. FL, full-length.

To identify antiviral cellular genes, we engineered replication-competent SIVcpz*Ptt* MB897 constructs carrying a compact (~351 nt) sgRNA expression cassette. As previously reported (13), this cassette contains the human U6 promoter and an sgRNA with a flexible targeting sequence and an invariant scaffold. All viral genes remain intact and are expressed under the native LTR and splice sites, while sgRNAs are independently transcribed from the U6 promoter (Fig. 2A). To preserve a functional *nef* gene, we eliminated its overlap with the U3 region of the viral 3′LTR and inserted the sgRNA-cassette between them. Previous analyses of HIV-1 constructs showed that duplicated *nef*/U3 sequences or direct repeats may drive loss of the cassette during viral replication (13). To circumvent this, we designed three SIVcpz*Ptt* variants: (i) Mut1, with codon-optimized *nef* gene to minimize homology with the U3 region and (ii) Mut2, that harbors a truncated 3′LTR U3 region but maintains critical *cis-acting elements* and the core enhancer/promoter elements. Notably, large parts of the U3 region serve mainly as Nef coding region and are dispensable for viral replication both *in vitro* and *in vivo* (22–24). (iii) Mut3 is similar to Mut2 but contains additional changes at the 3′end of *nef* to remove another potential recombination site (Fig. 2A).

Transfection of all proviral constructs yielded high levels of infectious SIVcpz*Ptt* MB897 (Fig. 2B), which replicated efficiently in SupT1 R5 Cas9 cells. However, RT-PCR analyses revealed that Mut1 largely lost the U6-sgRNA-scaffold cassette after 12 days, whereas Mut2 and Mut3 remained stable (Fig. 2C). Since Mut3 showed only minimal cassette loss, we selected it for generation of the SIVcpz-based library. As reported for HIV-1 (13), we generated SIVcpz*Ptt* TV constructs targeting 510 candidate antiviral genes, each by three distinct sgRNAs. Of these, 200 genes shared features of known antiviral factors (25), and the remaining 310 were chosen because of their potential roles in viral sensing or HIV-1 replication (26). Cloning was highly efficient and deep sequencing confirmed the presence of all 1,537 sgRNAs. Moreover, sgRNA frequencies in viral stocks closely matched those in the proviral TV-MB897-sgRNA library (Fig. 2D).

Since many well-known antiviral factors are IFN-inducible, we treated SupT1-R5 Cas9 cells with IFN-α or IFN-β. Both robustly induced ISG15 and BST-2 (tetherin), confirming activation of IFN-stimulated genes (ISGs, Fig. S2A through C). To identify both constitutive and IFN-induced antiviral factors, we initially cultured the TV-MB897-sgRNA library in the presence or absence of nontoxic concentrations of IFN-β (100 U/mL; Fig. S2D) for 12 days (each in two independent experiments). We then used the supernatants obtained at the end to initiate a second virus propagation period (Fig. S3). Conditions were modified compared to previous passaging of HIV-1 (13) because SIVcpz replicates with slower kinetics and has been reported to be more dependent on cell-to-cell spread (27, 28). Infection of TZM-bl cells with culture supernatants revealed higher infectious virus production during the second culture period (Fig. 2E), suggesting the potential emergence of SIVcpz variants with increased replication fitness. Unexpectedly, however, SIVcpz*Ptt* TV-MB897-sgRNA constructs consistently lost the sgRNA cassette in the presence of IFN-β at later time points (Fig. 2F). In contrast, they replicated efficiently and remained stable in the absence of IFN-β. Consequently, subsequent selection experiments and deep sequencing analyses were performed in the absence of IFN.

### Enrichment of sgRNAs targeting genes restricting SIVcpz*Ptt* replication

**Fig 3:**
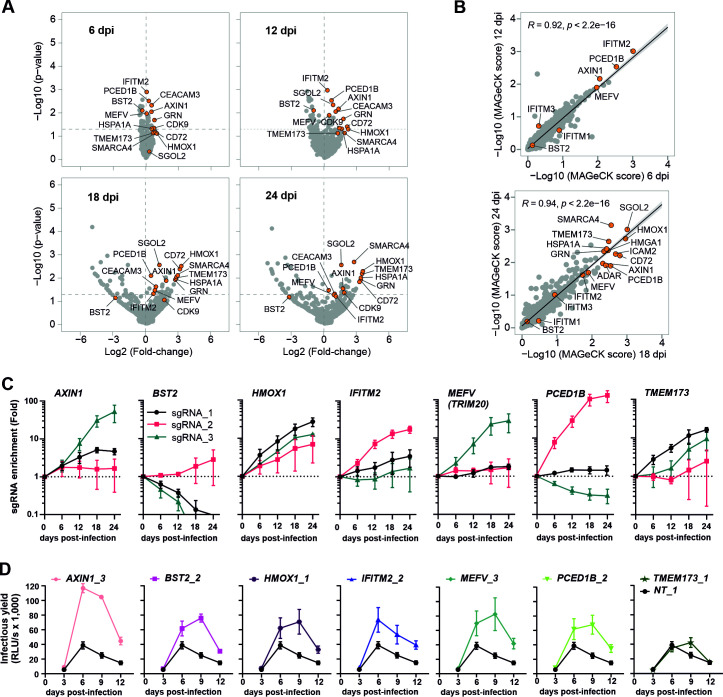
SIVcpz-driven enrichment and validation of sgRNAs. (**A**) Volcano plots indicating enrichment of specific genes targeted by sgRNAs during culture of the MB897-sgRNA library in SupT1-R5 Cas9 cells. (**B**) Correlation of gene-level MAGeCK enrichment scores at the indicated time-points post-infection. (**C**) Relative read counts from MAGeCK analysis showing enrichment of sgRNAs targeting *AXIN1, BST-2, HMOX1, IFITM2, MEFV, PCED1B,* or *TMEM173* during library propagation. (**D**) Validation of individual SIVcpz*Ptt* MB897 constructs expressing targeting sgRNAs or a non-targeting control in SupT1-CCR5 Cas9 cells. Numbers after the underscore specify the sgRNA. Infectious virus yield was quantified by TZM-bl assay. Data represent means of three independent experiments ± SEM.

To identify sgRNAs that promote SIVcpz*Ptt* replication, we analyzed virus-containing cell culture supernatants obtained in 6-day intervals from two independent cultures by next generation sequencing (NGS). MAGeCK analysis (29) allowed us to follow the frequency of all sgRNAs during virus propagation. Widening volcano plots revealed progressive enrichment or depletion of specific sgRNAs (Fig. 3A) with highly significant correlations across different timepoints (Fig. 3B). Although some top scoring hits varied between early and late time points, several candidates consistently emerged. At days 6 and 12, the most enriched genes included *IFITM2, PCED1B, AXIN1, CEACAM3,* and *MEFV*. At days 18 and 24, the top hits included SGOL2 (chromosome segregation) (30), SMARCA4 (chromatin remodeling) (31), TMEM173 (STING, a cytosolic DNA sensor) (32), and HMOX1 (a heme-degrading enzyme with anti-inflammatory effects). In agreement with the previous HIV-1 screen (13), sgRNAs varied in selection efficiency, but their relative effects on viral replication fitness were highly reproducible and, in most cases, at least two of three sgRNAs clearly enriched (Fig. 3C; Fig. S4A). Notably, it has been reported that pandemic HIV-1 M strains evolved a capsid that prevents cGAS and TRIM5 triggering (33). This may explain why sgRNAs targeting MAVS and STING were more efficiently selected in the context of the SIVcpz backbone. Altogether, hit factors may restrict SIVcpz through diverse mechanisms including modulation of innate immunity signaling and oxidative stress, interference with viral transcription and regulation of cell cycling.

To validate candidate antiviral factors, we generated eight individual SIVcpz*Ptt* MB897 constructs encoding sgRNAs targeting *AXIN1, BST-2, HMOX1, IFITM2, MEFV, PCED1B*, or *TMEM173* or a non-targeting (NT) control. Except for *BST-2* (tetherin), these targets were chosen based on their strong enrichment in the screens (Fig. 3A through C). Although SIVcpz cannot antagonize human tetherin (11, 12), it was not a significant hit (Fig. 3C), most likely because it is hardly expressed in untreated SupT1-R5 cells (Fig. S3B) (34). However, tetherin expression was strongly induced by IFNs (Fig. S3B) but only marginally by SIVcpz*Ptt* infection (Fig. S3C), which may explain the modest enhancing effect of just one of the three *BST-2* targeting sgRNAs (Fig. 3C). All targeting sgRNAs increased SIVcpz*Ptt* MB897 replication in SupT1-R5 Cas9 cells, albeit with different efficiency (Fig. 3D). While most sgRNAs enhanced infectious virus yields by ~2-fold, AXIN1 had very strong and TMEM173 (encoding STING) only marginal effects. Importantly, none of the sgRNAs significantly affected replication in parental SupT1-R5 cells lacking Cas9 (Fig. S4B). Altogether, these results show that sgRNAs targeting *AXIN1, BST-2, HMOX1, IFITM2, MEFV,* and *PCED1B* enhance SIVcpz*Ptt* replication in a Cas9/CRISPR-dependent manner in the human SupT1-R5 T cell line.

### Overlapping sets of sgRNAs increase replication fitness of SIVcpz and HIV-1

**Fig 4:**
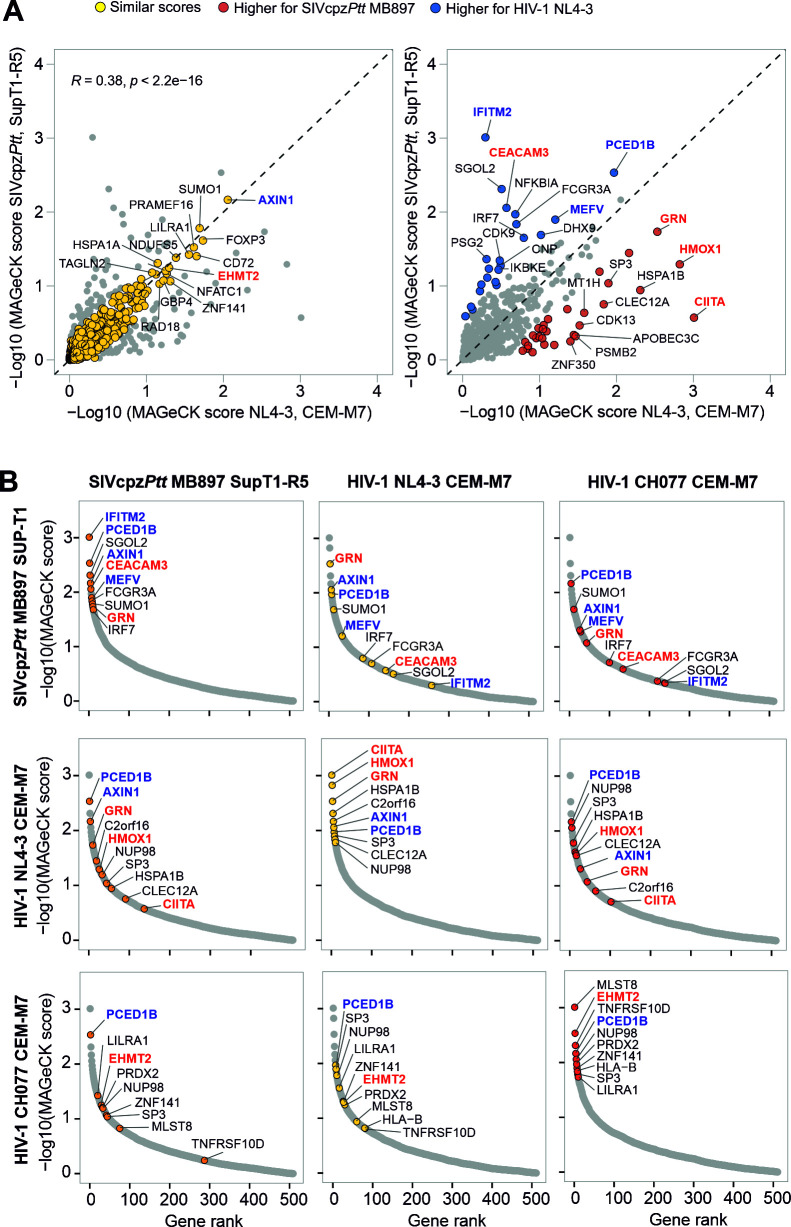
Overlaps and differences between sgRNAs enriched in SIVcpz- and HIV-1-driven screens. (**A**) MAGeCK scores obtained for each gene in the SIVcpz- and HIV-1-driven screens performed in SupT1-R5 and CEM-M7 Cas9 cells at 12 and 10 dpi, respectively. The left panel highlights genes with similar, and the right panel genes with different selection strengths by SIVcpz*Ptt* MB897 (red dots) or HIV-1 NL4-3 (blue dots). Factors selected for functional analyses are highlighted in blue and those analyzed in the previous HIV-1 screen in red. (**B**) Top 10 genes obtained in the SIVcpz and HIV-1 driven screens based on MAGeCK scores and their ranking in other screens at 12 dpi.

To compare the SIVcpz-driven screen with previous HIV-1 NL4-3 and CH077 data sets using the same sgRNAs library in CEM-M7 Cas9 cells (13), we performed correlation analyses. As expected, the strongest correlations arose between matching viral libraries at similar time points (Fig. S5). Several sgRNAs, including *AXIN1, SUMO1, FOXP3, PRAMEF16, CD72, NDUFS5, HSPA1A, EHMT2, NFATC1, TAGLN2, GBP4*, and *RAD18,* displayed consistent MAGeCK scores across SIVcpz and HIV-1, while others showed virus-specific enrichment (Fig. 4A). AXIN1 is a negative regulator of Wnt signaling (35). SUMO1 modulates numerous cellular processes and is known to enhance TRIM5α-mediated retroviral restriction (36). FOXP3, a master transcriptional regulator of regulatory T cells, has been reported to exert both inhibitory and enhancing effects on HIV-1 LTR-driven gene expression (37, 38). HSPA1A (Hsp70) had been reported to prevent Vpr-induced cell cycle arrest (39), suppress LTR transcription by displacing NF-κB p50/p65 (40), and inhibit Vif-mediated ubiquitination and degradation of APOBEC3G (41). Notably, *HSPA1B*, a close relative of *HSPA1A,* also emerged in the HIV-1-driven screen (Fig. 4A, right). EHMT2 (G9a) mediates H3K9 dimethylation and promotes HIV-1 latency in primary CD4+ T cells (42). NFATC1, a calcium-responsive transcription factor, may inhibit LTR transcription by competing with NF-κB for overlapping promoter binding sites (43). RAD18, a DNA repair factor, has been reported to protect target cells against HIV-1 infection (44). The roles of PRAMEF16, NDUFS5*,* TAGLN2, and GBP4 in HIV-1 replication are unknown. GRN, HMOX1, and CIITA were top hits in the HIV-1 screen and already confirmed to exert antiretroviral activity (13). Although CEACAM3 restricts HIV-1 replication in primary CD4+ T cells (13), it exhibited higher MAGeCK scores in the SIVcpz*Ptt* screen (Fig. 4A, right).

To further define factors enhancing viral replication in a species- or strain-specific manner, we compared MAGeCK score-based rankings of the top 10 genes from the SIVcpz*Ptt* MB897, HIV-1 NL4-3, and CH077 screens (Fig. 4B). Fourteen genes consistently ranked among the top 50 hits in all screens: *PCED1B, SGOL2, AXIN1, CEACAM3, MEFV, FCGR3A, SUMO1, GRN, EHMT2, PRDX2, SP3, LILRA1, HSPA1B,* and *HMOX1*. The corresponding cellular factors likely fall into several mechanistic clusters. Transcriptional repressors such as EHMT2, SP3, and SUMO1 may silence proviral gene expression through histone modification, SUMOylation, or NF-κB inhibition. Stress-response and antioxidant enzymes, like HMOX1, PRDX2, and HSPA1, likely dampen oxidative and inflammatory pathways that promote viral transcription and replication. Immune regulatory molecules (MEFV, CEACAM3, FCGR3A, LILRA1, and GRN) may modulate innate immune sensing or facilitate clearance of infected cells. Nuclear transport and signaling factors such as SGOL2 and AXIN1 could alter nuclear pore function or transcriptional cofactor availability. Consistent with previous reports that different HIV-1 strains may yield distinct ISG hits (45, 46), CIITA (class II major histocompatibility complex transactivator) ranked highly for NL4-3 but not for MB897 or CH077, whereas MLST8 (MTOR-associated protein, LST8 homolog) was specific to CH077 (Fig. 4B). Notably, overexpression of FCGR3A (Fc gamma receptor IIIa) has been previously shown to inhibit infectious HIV-1 production (25). Together, these results indicate that SIVcpz*Ptt* and HIV-1 select overlapping yet distinct sets of genes, with some shared targets differing in selection efficiency. We prioritized *IFITM2*, MEFV, *AXIN1*, and *PCED1B* for further analysis because sgRNAs targeting these genes were rapidly enriched and enhanced SIVcpz*Ptt* MB897 replication in SupT1 Cas9 cells (Fig. 3). In addition, their roles in lentiviral restriction are poorly understood, and *IFITM2*, MEFV, and *PCED1B* sgRNAs were more efficiently selected in the SIVcpz screen (Fig. 4).

### Endogenous MEFV, AXIN1, and IFITM2 restrict SIVcpz in SupT1-R5 cells

**Fig 5:**
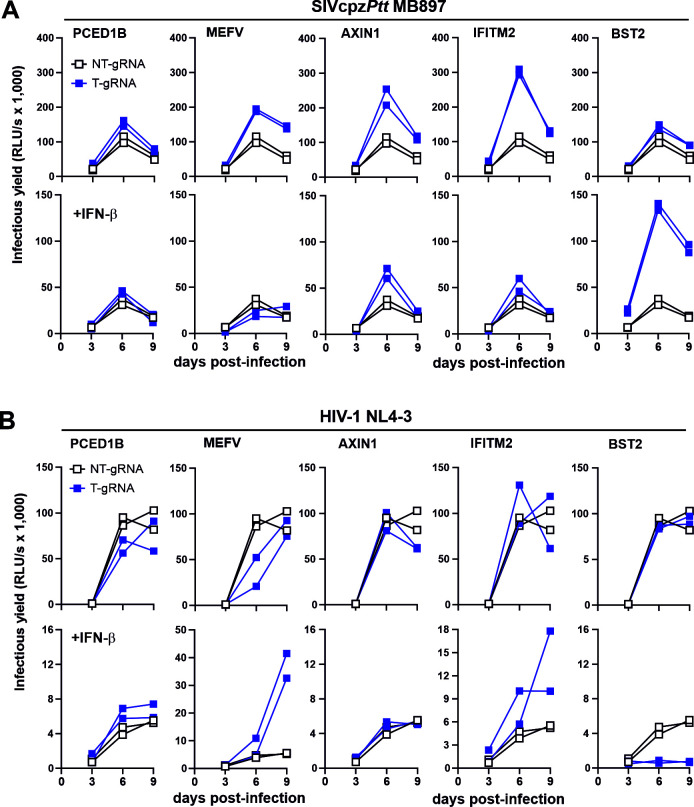
Effects of candidate KOs on SIVcpz*Ptt* MB897 and HIV-1 NL4-3 replication in SupT1-CCR5-Cas9 cells. (**A, B**) SupT1-CCR5 cells stably expressing Cas9 were electroporated with non-targeting (NT) gRNAs or gene-specific gRNAs targeting PCED1B, MEFV, AXIN1, IFITM2, or BST-2 followed by infection with (**A**) SIVcpz MB897 or (**B**) HIV-1 NL43 in the absence or presence of IFN-β. Shown are infectious virus yields quantified by TZM-bl infection assay over time from two independent experiments.

To verify the results obtained using sgRNA-expressing SIVcpz constructs, we performed conventional knockout (KO) analyses in SupT1-R5 cells. In the absence of IFN-β, disruption of MEFV, AXIN1, and IFITM2 increased infectious SIVcpz*Ptt* MB897 production by ~2- to 4-fold relative to NT controls but had no enhancing effects on HIV-1 NL4-3 replication (Fig. 5). IFN-β treatment strongly suppressed replication of both viruses. KO of BST-2 upon IFN-β treatment increased the levels of infectious SIVcpz*Ptt* MB897 in cell culture supernatants by ~5-fold (Fig. 5A) but prevented HIV-1 replication (Fig. 5B). Although the latter result appears counterintuitive, it is consistent with recent data showing that HIV-1 propagation in T cell lines requires basal levels of tetherin expression (47). In the presence of IFN-β, KOs had only modest (AXIN1, IFITM2) or no (PCED1B, MEFV) enhancing effects on SIVcpz*Ptt* MB897 infectious virus yields, presumably because high tetherin expression generally prevented efficient virion release (Fig. 5A). In comparison, KO of MEFS and IFITM2 clearly enhanced HIV-1 infectious virus yields in the presence of IFN-β (Fig. 5B). Altogether, these results show that the antiviral factors investigated have differential effects on SIVcpz and HIV-1 depending on expression levels and a potential dominant effect of BST-2 in the presence of IFN-β. Notably, basal expression of MEFV, AXIN1, and IFITM2 already restricted SIVcpz MB897 but not HIV-1 NL4-3.

### IFITM2 efficiently restricts SIVcpz*Ptt* and some HIV-1 group M strains

**Fig 6:**
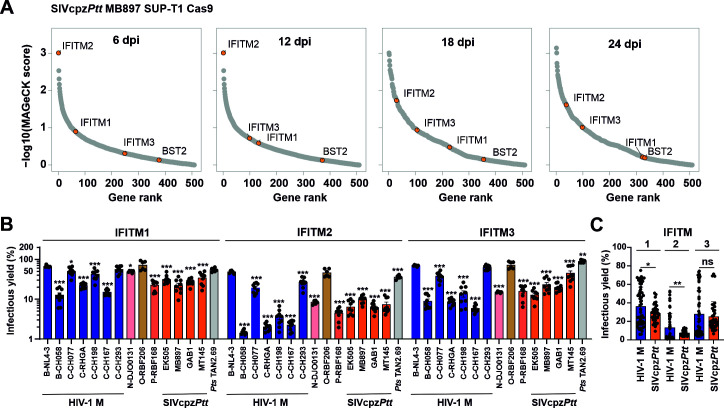
Enrichment of IFITM-targeting sgRNAs and antiviral activity of IFITMs. (**A**) Position of the three IFITM genes based on MAGeCK scores at the indicated days post-infection. BST-2 is shown for comparison. (**B**) HEK293T cells were transfected with IFITM expression vectors and the indicated HIV and SIV IMCs. Data show infectious virus yield relative to NT. *P* values are shown relative to those obtained for HIV-1 NL4-3. Dots represent data from three experiments each measured in duplicate or triplicate (mean ± SEM). (**C**) Average values (±SEM) obtained for all seven group M and the four SIVcpz*Ptt* strains. *P* values were determined using a two-tailed Student’s *t*-test with Welch’s correction. Significant differences are indicated as *P < 0.05; **P < 0.01; ***P < 0.001; ns, not significant.

IFITM3 is a well-established inhibitor of HIV-1 entry that blocks fusion of viral and host membranes (48–50). IFITM1 and IFITM2 are less well characterized and commonly considered weaker restriction factors. Unexpectedly, however, sgRNAs targeting *IFITM2* were enriched more efficiently than those targeting *IFITM3* (Fig. 6A). SIVcpz*Ptt* MB897 expressing an IFITM2-targeting sgRNA outcompeted an otherwise isogenic virus containing NT sgRNA (Fig. 3C) and produced higher levels of infectious virus in SupT1-R5 Cas9 cells (Fig. 3D). Overexpression of IFITM2 in HEK293T cells inhibited SIVcpz*Ptt* MB897 in a dose-dependent manner, while AXIN1, MEFV, and PCED1B had little or no effect (Fig. S6). This agrees with findings that IFITMs incorporate into virions and reduce infectivity (51). To directly compare antiviral potency, HEK293T cells were cotransfected with IFITM1, IFITM2, or IFITM3 expression vectors and a panel of 15 different HIV-1 and SIVcpz IMCs. Western blots confirmed similar expression levels of different IFITMs (Fig. S7). Consistent with the results of the screen, IFITM2 showed the strongest antiviral activity (Fig. 6B), reducing infectious yields of HIV-1 M strains by an average of 85% and four SIVcpz*Ptt* IMCs by 92%, compared to 62% and 72% for IFITM1 and 70% and 75% for IFITM3, respectively (Fig. 6C).

On average, SIVcpz*Ptt* strains were significantly more sensitive to IFITMs than HIV-1 IMCs (Fig. 6C). However, the sensitivity of HIV-1 group M strains varied substantially. Transmitted-founder HIV-1 IMCs CH058, RHGA, CH198, and CH167 were highly sensitive, while NL4-3, CH077, and CH293 were more resistant. HIV-1 N DJO0131 was also sensitive, whereas HIV-1 O RBF206 and SIVcpz*Pts* TAN2.69 were largely resistant against overexpression of IFITMs. Notably, SIVcpz*Ptt* MB897 was more sensitive to IFITM-mediated restriction than HIV-1 NL4-3 and CH077 used in prior screens (13), which agrees with stronger enrichment of IFITM2-targeting gRNAs by SIVcpz*Ptt* (Fig. 4). Altogether, the results show that IFITM2 overexpression in virus-producer cells inhibits SIVcpz*Ptt* more efficiently than IFITM1 and IFITM3. HIV-1 strains differ substantially in IFITM susceptibility, and the relative resistance of NL4-3 may explain why IFITM2’s antiviral activity has been underestimated in previous studies.

### Increased AXIN1 expression suppresses SIVcpz*Ptt* and HIV-1 replication

**Fig 7:**
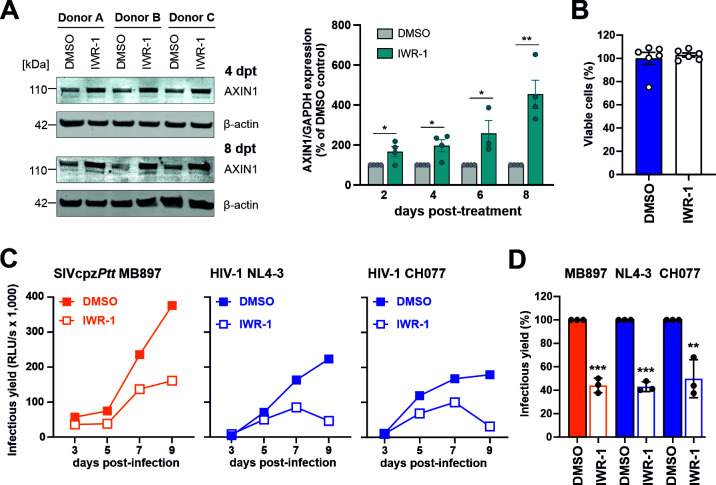
IWR-1 enhances AXIN1 expression and restricts viral replication. (**A**) Primary CD4^+^ T cells from healthy donors were treated every 2 days with 10 µM IWR-1 (dissolved in DMSO) or DMSO alone. Cells were harvested at the indicated time points for western blot analysis. The left panel shows AXIN1 expression in a representative donor at 4 and 8 days post-treatment. The right panel shows mean ± SEM percentages of AXIN1 expression in IWR-1-treated cells relative to DMSO controls (set to 100%). Statistical significance was determined using an unpaired t test (*P < 0.05; **P < 0.01). (**B**) Uninfected primary CD4^+^ T cells were treated with 10 µM IWR-1 or DMSO, and cell viability was assessed 2 days post-treatment using the CellTiter-Glo Luminescent Cell Viability Assay. (**C**) Primary CD4^+^ T cells were infected with SIVcpzPtt MB897, HIV-1 NL4-3, or CH077 and treated every 2 days with 10 µM IWR-1 or DMSO. Supernatants collected at 3, 5, 7, and 9 days post-infection were analyzed by TZM-bl assay. Data show representative replication kinetics from one of three donors, measured in two biological replicates. (**D**) Cumulative viral production from all three donors, normalized to DMSO controls (100%). *P* values were determined using a two-tailed Student’s *t*-test with Welch’s correction: **P < 0.01; ***P < 0.001.

AXIN1 (Axis Inhibition Protein 1) emerged as a hit in both SIVcpz*Ptt*- and HIV-1-driven screens (Fig. 4), and sgRNAs targeting *AXIN1* strongly enhanced SIVcpz replication (Fig. 3). However, AXIN1 overexpression in HEK293T cells failed to inhibit SIVcpz*Ptt* MB897 and HIV-1 NL4-3 (Fig. S6). To further examine whether AXIN1 restricts viral replication in a cell type specific manner, we infected primary CD4+ T cells with SIVcpz*Ptt* MB897 and HIV-1 NL4-3 or CH077 in the presence of 10 µM IWR-1, a tankyrase inhibitor that stabilizes AXIN1 (52, 53). Treatment with IWR-1 significantly enhanced the levels of AXIN1 expression (Fig. 7A) without causing cytotoxic effects (Fig. 7B). IWR-1 treatment inhibited replication of SIVcpz*Ptt*, as well as both HIV-1 strains (Fig. 7C) and reduced infectious virus production by ~50%–60% (Fig. 7D). These findings indicate that artificially increased AXIN1 activity restricts both SIVcpz*Ptt* and HIV-1 replication in primary T cells.

### Host factors restricting SIVcpz*Ptt* but not HIV-1 in primary CD4^+^ T cells

**Fig 8:**
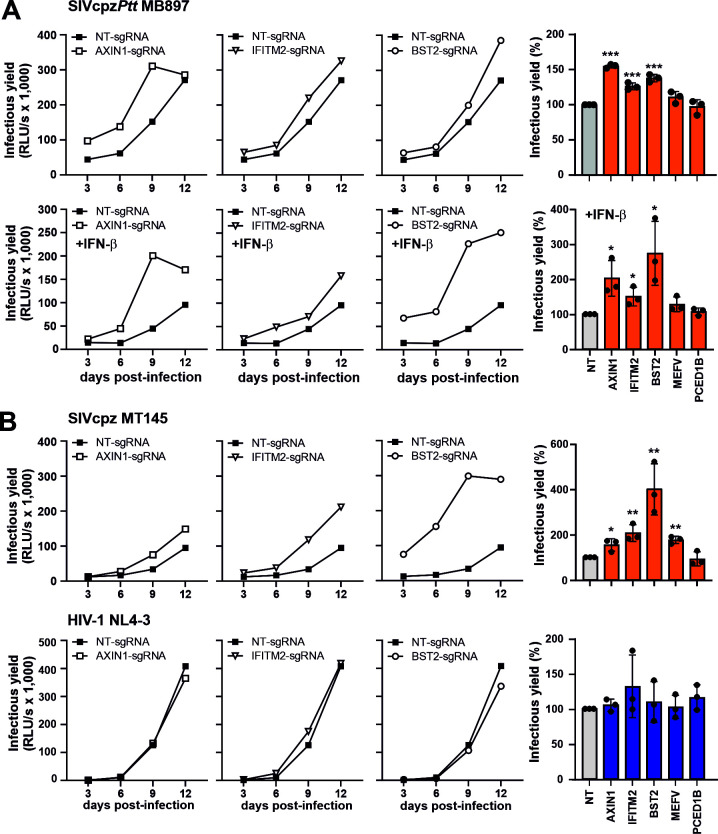
Effects of AXIN1, IFITM2, and tetherin KO on SIVcpz*Ptt* and HIV-1. (**A**) Primary CD4+ T cells were electroporated with AXIN1-, IFITM2-, BST-2-, or non-targeting (NT) sgRNAs followed by infection with SIVcpz*Ptt* MB897 in the absence (upper) or presence (lower) of IFN-β. Left panels: replication kinetics in cells from a representative donor. Each point represents the mean infectious virus yield from triplicate measurements. Right panels: cumulative infectious virus production by three donors, normalized to NT controls (100%). (**B**) Same as in panel A, except that cells were infected with SIVcpz*Ptt* MT145 or HIV-1 NL4-3. Only results in the absence of IFN are shown since replication in presence of IFN-β was too low for meaningful analysis. *P* values were determined using a two-tailed student’s *t*-test with Welch’s correction. Significant differences are indicated as *P < 0.05; **P < 0.01; ***P < 0.001.

To assess the potential *in vivo* relevance of factors identified by the SIVcpz*Ptt* TV screen, we confirmed expression of IFITM2, AXIN1, MEFV, and PCED1B in primary CD4+ T cells (Fig. S8). For comparison, we also included BST-2. To further examine the antiviral activity of these factors, we used a previously established sgRNA/Cas9-based KO approach in primary CD4^+^ T cells (13). KO efficiently reduced protein levels of IFITM2, BST-2, and AXIN1, while depletion of MEFV and PCED1B was inefficient (Fig. S8). Reduced AXIN1, IFITM2, and BST-2 expression significantly enhanced SIVcpz*Ptt* MB897 replication, with the effect of BST-2 being stronger in the presence of IFN-β (Fig. 8A). To confirm inhibition of SIVcpz and examine susceptibility of HIV-1, we analyzed a second SIVcpz*Ptt* IMC (MT145) (15) and HIV-1 NL4-3. Partial KO of *IFITM2*, *AXIN1, MEFV,* and (most strongly) *BST-2* significantly increased replication of SIVcpz*Ptt* MT145 but had no enhancing effect on HIV-1 NL4-3 (Fig. 8B). Altogether, the results revealed that endogenous levels of AXIN1, IFITM2, MEFV, and BST-2 restrict SIVcpz replication in primary human CD4+ T cells, whereas HIV-1 NL4-3 is resistant.

**Fig 9:**
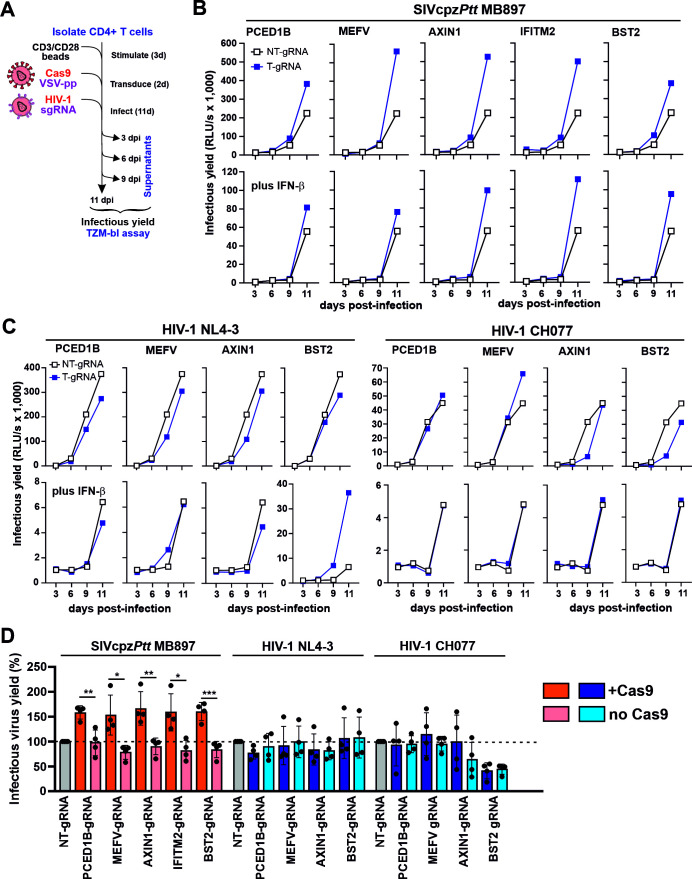
Effects of sgRNAs on SIVcpz and HIV-1 replication in Cas9-expressing CD4^+^ T cells. (**A**) Experimental schematic. (**B**) Replication of SIVcpz*Ptt* MB897 constructs encoding sgRNAs targeting the indicated genes in Cas9-expressing primary CD4^+^ T cells. Infectious virus yields were measured by TZM-bl assay. Shown are representative results for one donor, and similar results were obtained in cells from three different additional blood donors. (**C**) Replication of HIV-1 constructs encoding the same sgRNAs as in panel B. Panels B and C show data from the same representative donor as in panel A. (**D**) Cumulative infectious yields from Cas9-expressing or control CD4^+^ T cells from four donors, normalized to the non-targeting (NT) sgRNA control (100%). Data represent means ± SEM from four independent experiments. *P* values were calculated using two-tailed student’s *t*-tests with Welch’s correction. Significance: *P < 0.05; **P < 0.01; ***P < 0.001.

Conventional KO strategies, like the ones above, are limited for studying antiviral factors because they alter cells before infection, potentially obscuring factors with distinct effects at different stages of viral replication, such as CD4 or NF-κB. In addition, they may impact cell viability, especially since non-infected bystander cells are also affected. Furthermore, KO of MEFV and PCED1B was inefficient (Fig. S8). To overcome these issues, we transduced stimulated primary CD4^+^ T cells from four independent donors with a VSVg-pseudotyped lentiviral vector expressing Cas9 (Fig. 9A; Fig. S9). Two days later, these cell cultures were infected with SIVcpz*Ptt* MB897 constructs encoding sgRNAs against *IFITM2, AXIN1, MEFV, PCED1B*, or *BST-2*, or a non-targeting control. All five targeting sgRNAs enhanced replication of SIVcpz*Ptt* MB897 in both the absence and presence of IFN-β (Fig. 9B), and this enhancement was dependent on Cas9 expression (Fig. S10A). These results suggest that IFITM2, AXIN1, MEFV, PCED1B, and BST-2 restrict SIVcpz replication in primary human CD4+ T cells at both basal and IFN-inducible expression levels. In contrast, analogous HIV-1 NL4-3 and CH077 constructs carrying sgRNAs against *AXIN1, MEFV, PCED1B*, or *BST-2* did not show increased replication (Fig. 9C; Fig. S10B). On average, sgRNAs targeting all five factors identified by the SIVcpz-driven CRISPR screen significantly increased infectious SIVcpz*Ptt* MB897 production compared to the NT control, while none increased HIV-1 replication (Fig. 9D). Together, these results demonstrate that the TV approach identifies host factors that restrict SIVcpz*Ptt*, but not HIV-1 group M strains, in primary human CD4^+^ T cells.

## DISCUSSION

Only one of at least four zoonotic transmissions of simian immunodeficiency viruses from chimpanzees (SIVcpz) or gorillas (SIVgor) gave rise to the AIDS pandemic (8, 9). Identifying human host factors that restricted SIVcpz provides insights into the evolutionary barriers HIV-1 group M strains overcame to spread efficiently in humans. Using a replication-competent SIVcpz-driven CRISPR–Cas9 screen, we identified a variety of host genes including *IFITM2*, *AXIN1, MEFV,* and *PCED1B* that restrict SIVcpz*Ptt* MB897 replication in human SupT1 cells. Functional analyses confirmed that these factors inhibit SIVcpz*Ptt* but not HIV-1 replication in primary human CD4+ T cells. These findings reveal host defense mechanisms that likely shaped lentiviral evolution and the emergence of pandemic HIV-1.

IFITM2 emerged as a top hit in the SIVcpz*Ptt*-driven CRISPR screen (Fig. 4B). IFITMs are known to be incorporated into budding virions and reduce HIV-1 infectivity (49, 51, 54, 55). However, it was unexpected that sgRNAs targeting IFITM2 were selected more efficiently than those targeting IFITM3 (Fig. 5A) since it is commonly thought that the antiviral potency ranks IFITM3 > IFITM2 > IFITM1 (51, 56). Consistent with the results of the CRISPR screens, overexpression of IFITM2 reduced the infectious yields of diverse HIV-1 and SIVcpz IMCs more efficiently than IFITM3 (Fig. 5B and C). On average, SIVcpz*Ptt* IMCs were more susceptible to IFITM2 restriction than HIV-1 group M strains, but the sensitivity of the latter varied considerably. Notably, NL4-3 and CH077 used in our previous CRISPR screens were largely resistant, whereas some TF HIV-1 IMCs showed marked sensitivity to IFITM2 and IFITM3 restriction (Fig. 5B). This was unexpected since it has been reported that TF HIV-1 strains are relatively resistant to IFITM-mediated restriction (50). Altogether, the relative antiviral potency of IFITMs is dependent on both the assay and the viral context. Most previous studies relied on IFITM-overexpressing cell lines, whereas our data show that IFITM2 restricts SIVcpz*Ptt* MB897 and MT145, but not HIV-1 NL4-3, in primary human T cells. These findings support a role of IFITM2 in innate defense against SIVcpz*Ptt* and suggests potential adaptation to the human host for efficient viral transmission. It has recently been reported that Nef counteracts HIV-1 restriction by IFITM2 and IFITM3 (57), and it will be of interest to clarify whether Nef determines primate lentiviral sensitivity to IFITM proteins. Notably, SIVcpz*Pts* TAN2.69 from eastern chimpanzees was largely resistant to IFITM overexpression (Fig. 5B). Thus, higher susceptibility of SIVcpz*Pts* to IFITMs does not explain why only SIVcpz*Ptt* has been detected in humans.

AXIN1, a central scaffold protein in the Wnt signaling pathway (35), was identified as another inhibitor of SIVcpz*Ptt* replication. AXIN1-targeting sgRNAs significantly increased SIVcpz*Ptt* MB897 replication in both SupT1 cells and primary human CD4+ T cells in a Cas9-dependent manner (Fig. 3D and 8). In contrast, overexpression of AXIN1 in HEK293T cells had little effect on infectious virus yields (Fig. S6), suggesting a context-dependent function that may be specific to T cells. Previous studies on HIV-1 reported that Wnt signaling inhibits viral replication and promotes viral latency in a β-catenin dependent manner (58, 59). AXIN1 is a negative regulator of the Wnt pathway (35). Thus, its identification as an effective inhibitor of SIVcpz*Ptt* was unexpected. However, it agrees with recent data showing that AXIN1 boosts the antiviral response through IRF3 stabilization (60). AXIN1-targeting sgRNAs were enriched in the context of both SIVcpz*Ptt* and HIV-1 (Fig. 4) and artificially enhanced AXIN1 expression inhibited replication of both SIVcpz and HIV-1 (Fig. 7). In contrast, endogenous AXIN1 expression inhibited replication of SIVcpz*Ptt* but not HIV-1 in primary CD4+ T cells (Fig. 9) indicating that HIV-1 evolved to evade restriction by normal physiological levels in its primary target cells but not AXIN1 activity that is artificially increased by IWR-1. Together, these findings identify AXIN1 as a T cell-specific antiviral factor for SIVcpz*Ptt* and suggest that reduced susceptibility to AXIN1-mediated inhibition facilitates efficient spread of HIV-1 in humans.

MEFV (Mediterranean fever gene) and PCED1B (PC-esterase domain containing 1B) were also identified as inhibitors of SIVcpz*Ptt* MB897 replication. In SupT1-R5 Cas9 cells, sgRNAs targeting these genes significantly enhanced viral replication (Fig. 3D), suggesting roles in the innate antiviral defense. MEFV encodes pyrin, a regulator of inflammasome activity and inflammatory signaling, that has been implicated in controlling human pathogens and in induction of autophagy and autoinflammatory diseases (61–64). Further studies are required to determine whether its antiviral activity involves inflammasome-mediated restriction or modulation of cellular stress responses during HIV-1 infection. PCED1B is less well characterized, but its predicted esterase activity suggests it may influence lipid metabolism or membrane composition, potentially impacting viral assembly or egress. Overexpression of MEFV or PCED1B in HEK293T cells did not reduce infectious viral yields (Fig. S6), suggesting that their antiviral effects may be cell-type specific. Indeed, MEFV and PCED1B targeting sgRNAs significantly enhanced replication of SIVcpz*Ptt* but not of HIV-1 in primary human CD4+ T cells (Fig. 9; Fig. S10). These findings identify MEFV and PCED1B as novel antiviral factors for SIVcpz*Ptt* and suggest that resistance to their antiviral effects may have contributed to the successful adaptation of HIV-1 to humans.

Acquisition of Vpu as an effective tetherin antagonist was a major adaptation of HIV-1 group M to the human host and most likely played a key role in its pandemic spread (8). Nevertheless, tetherin was not among the top hits in our SIVcpz*Ptt*-driven CRISPR screen (Fig. 3). In part, this may reflect its low expression in SupT1 cells and the virus’s preferential cell-to-cell transmission mode (27, 28). However, it also indicates a limitation of our approach—its reliance on the efficiency and specificity of individual sgRNAs. Tetherin received a low MAGeCK score because only one of three targeting sgRNAs was enriched (Fig. 3C) most likely because SupT1 cells hardly express BST-2 in the absence of IFN. Notably, BST-2-targeting sgRNA enhanced SIVcpz*Ptt* MB897 replication in primary CD4+ T cells in a Cas9-dependent manner (Fig. 9B). In contrast, it did not enhance replication of HIV-1 NL4-3 and CH077, whose Vpu proteins efficiently antagonize human tetherin. A striking observation was the Cas9-independent enhancement of NL4-3 replication by the tetherin-targeting sgRNA in the presence of IFN-β (Fig. 9C). This increase was consistently observed in CD4^+^ T cells from all four donors (Fig. S10B) and warrants further investigation. Altogether, these findings confirm that human tetherin restricts SIVcpz but not HIV-1 and illustrate that inefficient sgRNAs may lead to underestimation or omission of bona fide restriction factors. However, continuous improvement of CRISPR-KO libraries, now achieving ~70% highly active sgRNAs, >95% gene coverage, and <5% off-target activity (65) will minimize this limitation. For most of our identified hits, at least two independent sgRNAs were significantly enriched during SIVcpz*Ptt* propagation (examples in Fig. 3C).

Since our screen is driven by viral replication fitness, with effects amplified over multiple rounds of replication, it is highly sensitive and robust. A major advance of the present study is that stable lentiviral delivery of Cas9 expression vectors into primary CD4+ T cells enabled direct testing of enriched sgRNAs in the main target cells of SIV and HIV-1. This system verified that endogenous expression of PCED1B, IFITM2, AXIN1, MEFV, and BST-2 restricts SIVcpz*Ptt* MB897 but not HIV-1 NL4-3 and CH077 in primary CD4+ T cells. While further studies are required to assess the variability of diverse SIVcpz and HIV-1 strains in susceptibility to inhibition, these data provide proof-of-concept that the TV approach allows to identify antiviral mechanisms that HIV-1 M has cleared during adaptation to humans. As also noted in our previous study (13), most factors limiting viral replication fitness were not ISGs. Antiviral factors that are constitutively expressed may represent the under-investigated real first line of defense as they do not require viral replication and innate immune activation to exert protective effects.

The TV approach offers many prospects for further studies. Constitutive or conditional Cas9-expressing mouse models are already in routine use, and the development of analogous non-human primate models is technically feasible (66). Thus, screening of TV-sgRNA libraries or verification of individual sgRNA in animal models for HIV/AIDS offers interesting perspectives. Notably, the TV approach not only identifies specific antiviral factors but also host genes affecting cellular pathways and general features that are not favorable for effective viral replication. Thus, genome-wide TV screens using different viral backbones, cell types, and experimental conditions should provide unprecedented deep new insights into the virus-host interplay.

## MATERIALS AND METHODS

### Phylogenetic analyses

Evolutionary analyses were conducted using NGPhylogeny (67, 68). Whole genome sequences (SIVcpz*Ptt* MB897: EF535994, SIVcpz*Ptt* MB66: DQ373063, SIVcpz*Ptt* LB7: DQ373064, HIV-1 M-B CH058.tf: JN944907, HIV-1 M-B CH077.tf: JN944941, HIV-1 M-B RHGA.cc: KC312535, HIV-1 M-B NL4−3 X4: AF003887, HIV-1 M-C CH198.tf: KC156130, HIV-1 M-C CH167.cc: KC156213, HIV-1 M-C CH293.cc: KC156216, HIV-1 N DJO0131: AY532635, SIVcpz*Ptt* EK505: DQ373065, SIVcpz*Ptt* MT145: DQ373066, SIVcpz*Ptt* GAB1: X52154, SIVcpz*Ptt* GAB2: AF382828, SIVcpz*Ptt* US: AF103818, SIVcpz*Ptt* CAM3: AF115393, SIVcpz*Ptt* CAM5: AJ271369, SIVcpz*Pts* TAN1: AF447763, SIVcpz*Pts* TAN2.69: DQ374657, SIVcpz*Pts* TAN3.1: DQ374658, SIVcpz*Pts* ANT: U42720, SIVgor CP684: FJ424871, HIV-1 N 04CM-1015-04: DQ017382, HIV-1 N YBF30: AJ006022, HIV-1 O MVP5180: L20571, HIV-1 O ANT70: L20587, HIV-1 O RBF206: KY112585, HIV-1 P 06CMU14788: HQ179987, HIV-1 P RBF168: GU111555) were obtained from the NCBI database and aligned using MAFFT. A phylogenetic tree based on sequence similarity is shown. Scale bar: 0.1 amino acid replacements per site. The phylogenetic tree showing distance-based relationship interference data based on nucleotide sequences was generated using the NGPhylogeny.fr FastME tool (https://ngphylogeny.fr/) and was visualized using iTOL (https://itol.embl.de/). The tree is drawn to scale, with branch lengths measured in the number of substitutions per site.

### Replication-competent SIVcpz*Ptt* MB897 sgRNA constructs

Generation of the pCR-XL-TOPO-SIVcpz MB897 IMC has been previously reported (15). To generate replication-competent SIVcpz-MB897 containing an sgRNA cassette, the cassette was inserted between the *nef* gene and a duplicated TPI/U3 sequence, which usually overlaps *nef*. To prevent recombination, the TPI and overlapping *nef* regions were codon-optimized (Mut1). To reduce genome size, partial upstream U3 sequences (primarily encoding Nef and partially for transcription regulation) were removed (22), retaining TPI and either 89 bp (Mut2) or 111 bp (Mut3) of U3 upstream of the TATA box.

### Construction of traitor SIVcpz*Ptt* MB897 proviral vectors

DNA segments containing human codon optimized *nef*, sgRNA expression cassette, and truncated/duplicated 3′LTRs were synthesized by Twist Bioscience, PCR amplified using Forward primer: 5′-AAGGAG-TAGGGCCAGTCTCG-3′ and Reverse primer: 5′-CCTCTAGATGCATGCTCGAGC-3′, and cloned into the *NruI/NotI* digested pCR-XL-TOPO-SIVcpz MB897 backbone using Gibson Assembly. Products were transformed into XL2-blue MRF' competent cells. All constructs were confirmed by Sanger sequencing in Microsynth Seqlab .

### Cloning of sgRNA library into traitor SIVcpz MB897 sgRNA cassette

To construct the traitor SIVcpz*Ptt* MB897 library, a DNA oligonucleotide library targeting 510 human genes (13) was synthesized by Twist Bioscience. The synthesized DNA oligonucleotides were amplified by polymerase chain reaction (PCR) using NEBNext High-Fidelity 2×PCR Master Mix (NEB, M0541L) and purified using the Monarch PCR & DNA Cleanup Kit (NEB, T1030L). The traitor SIVcpz*Ptt* MB897 vector was digested with Esp3I (Thermo Fisher Scientific, FD0454), and the amplified sgRNA sequences were assembled into the vector backbone with the Gibson Assembly method. Ligation products were purified using the Monarch PCR & DNA Cleanup Kit (NEB, T1030L). Two hundred nanograms of the purified ligation products was electroporated into 25 μL of Endura Competent Cells (Lucigen, 60242). Approximately 2.5 × 10^6^ recombinants were obtained, which yielded 1,500× library coverage. Bacteria were harvested, and the sgRNA library plasmids were extracted using the QIAGEN Plasmid Maxi Kit (Qiagen, 12165).

### Transfection and production of viral stocks

HEK293T cells were transiently transfected using PEI 25K (Polysciences, 23966-100) at a ratio of 2 μg of PEI per 1 μg of DNA, and the medium was replaced 4–6 h post-transfection. To test antiviral effects of potential restriction factors, pcDNA-based expression constructs were cotransfected with the proviral constructs. In titration experiments, empty vector was used to keep the total DNA amount constant. The transfected cells were incubated for 4–6 h before the medium was replaced by fresh DMEM with 2% FCS. To generate virus stocks, one day before transfection, 20 mio cells were seeded in 15 cm dishes in 20 mL medium to obtain a confluence of 80%–90% at the time of transfection. For transfection, 40 μg of DNA was mixed with 80 μg PEI 25K, incubated 15 min at RT, and added dropwise to the cells. Forty-eight hours post-transfection, the virus was harvested, centrifuged 5 min at 2,000 rpm, and concentrated 40 times using Amicon Ultra 15 mL Filters 100 kDa (Merck, UFC910096). The concentrated viruses were aliquoted and stored at −80°C.

### Cell culture

All cells were cultured at 37°C in a 5% CO_2_ atmosphere. Human embryonic kidney 293T cells purchased from American Type Culture Collection (ATCC: CRL3216) were cultivated in Dulbecco’s modified Eagle medium (DMEM, Gibco) supplemented with 10% (vol/vol) heat-inactivated fetal bovine serum (FBS, Gibco), 2 mM L-glutamine (PANBiotech), 100 μg/mL streptomycin (PANBiotech), and 100 U/mL penicillin (PANBiotech). TZM-bl cells were provided and authenticated by the NIH AIDS Reagent Program, Division of AIDS, NIAID, NIH from Dr. John C. Kappes, Dr. Xiaoyun Wu and Tranzyme Inc. THP-1 (ATCC, TIB-202), U-937 (ATCC, CRL-1593.2), PM1 (NIH AIDS Reagent Program, ARP-3038), Jurkat T4 (CD4^+^ human leukemia T cells) (ATCC, TIB-152), SupT1 CCR5 high Cas9 (13), and THP-1 Cas9 cells were cultured in Roswell Park Memorial Institute (RPMI) 1640 medium supplemented with 10% (vol/vol) heat-inactivated fetal bovine serum, 2 mM L-glutamine, 100 µg/mL streptomycin, 100 U/mL penicillin. For selection and maintenance of transgene expression, 0.3 μg/mL puromycin and 10 μg/mL blasticidin S HCl were added to the SupT1 CCR5 high Cas9 culture medium to maintain CCR5 and Cas9 expression, respectively. THP-1 Cas9 cells were maintained with 10 μg/mL blasticidin S HCl to retain Cas9 expression.

### Infection, kinetics, and traitor virus enrichment

To start the replication kinetic, 5 million SupT1 CCR5 Cas9 cells were infected with the traitor SIVcpz*Ptt* MB897 library constructs with the EF-C fibril enhancer (69) at a final concentration of 10 μg/mL. On the next day, cells were washed three times with PBS and seeded in T25 flask at a cell density of 0.7 million/mL in the presence or absence of 100 U/mL IFN-β (R&D Systems, 8499-IF-010). Every 2–3 days, cells were sub-cultured until 12 days post infection. At 12 d p.i., supernatants containing viruses were concentrated by Amicon Ultra 15 mL Filters 100 kDa, to infect fresh SupT1 CCR5 Cas9 cells as above, and cultured for another 12 days. Every 6 days, supernatants were collected and concentrated for further analysis. Kinetics were monitored by infecting TZM-bl cells.

### Detection of sgRNA cassette stability by reverse transcription PCR

To check the stability of the sgRNA cassette in the viral genome during passaging, viral RNA was isolated at different time points from supernatants with the QIAamp Viral RNA Mini Kit (Qiagen, 52906). Residual genomic DNA was digested and cDNA was synthesized using the PrimeScript RT Reagent Kit with gDNA Eraser (Takara, RR047A) according to the manufacturer’s instructions and specific primer 5′-GGGCAAGCCACTCCCTACC-3′. The cassette was amplified from the cDNA by KOD ONE DNA polymerase (TOYOBO, KMM-201NV) using Forward primer: 5′-GGATGGCCTGCAGTAAGG-GAC-3′, Reverse Primer: 5′-GGGCAAGCCACTCCCTACC-3′. PCRs were loaded onto a 1% agarose and ran at 140 V for 30 min.

### Viral RNA preparation for sequencing

Viral RNA was isolated from concentrated supernatants collected from the traitor SIVcpz*Ptt* MB897 library infected cells at indicated time points using the Viral RNA Mini Kit (Qiagen) according to the manufacturer’s instructions. Genomic DNA was digested, and cDNA was synthetized using the PrimeScript RT Reagent Kit with gDNA Eraser (Takara #RR047A) according to the manufacturer’s instructions and specific primer 5′- TAAAAAGTGGCTAGCGATCGC-3′. Twelve cDNA reactions for one sample were purified using the Monarch PCR Purification Kit (NEB, T1030L) and eluted in 20 μL ddH_2_O. The sgRNA cassette was amplified using the NEBNext High-Fidelity 2× PCR Master Mix (NEB) and primers including Illumina adapters and 8-nt barcodes to allow Next-Generation Sequencing analysis. PCRs were purified using the Monarch PCR & DNA Cleanup Kit (NEB, T1030L) and eluted in 20 μL ddH_2_O. Next-generation sequencing NGS was performed using the Illumina NextSeq2000 platform with 60 base-pair paired-end runs. Raw reads were demultiplexed on the Galaxy version 23.0 platform, and forward and reversed reads were merged with SeqPrep 0.2.2. Merged reads were trimmed by Cutadapt 4.4 and then aligned to the custom library sequences using the MAGeCK algorithm suite (Version 0.5.9.2.4). Individual read counts are determined and median-normalized for the effect of library sizes and read count distributions. Individual sgRNAs targeting the same gene are summarized, and a variance model calculated using a negative binomial model to statistically assess the difference between control (input) and the conditions (different days). Targets are ranked by MAGeCK according to their *P*-value via a modified robust ranking aggregation (RRA) algorithm (α-RRA) to identify enriched genes (Table S1). Overrepresented sgRNA sequences compared to the input control represent viruses carrying an sgRNA targeting a gene that restricts viral replication. Scatter plots were generated using R version 4.2.3, ggplot2 version 3.4.3, and smplot2 version 0.2.4.

### Viral infectivity

To determine infectious virus yield, 8,000 TZM-bl reporter cells/well were seeded in 96-well plates and infected with cell culture supernatants in triplicates on the following day. Three days post-infection, cells were lysed and β-galactosidase reporter gene expression was determined using the X-Gal Screen Kit (Applied Bioscience, T1027) according to the manufacturer’s instructions with an Orion microplate luminometer (Berthold).

### Supernatants and whole cell lysates

To determine expression of cellular and viral proteins, cells were washed in PBS and subsequently lysed in Western blot lysis buffer (150 mM NaCl, 50 mM HEPES, 5mM EDTA, 0.1% NP40, 500 μM Na_3_VO_4_, 500 μM NaF, pH 7.5) or radioimmunoprecipitation assay (RIPA) buffer (50 mM Tris-HCl; pH 7.4, 150 mM NaCl, 1% [vol/vol] NP-40, 0.5% [wt/vol] deoxycholic acid [DOC], 0.1% [wt/vol SDS]) supplemented with protease inhibitor (Roche, 1:500). After 5 min of incubation on ice, samples were centrifuged (4°C, 20 min, 14,000 rpm) to remove cell debris. The supernatant was transferred to a fresh tube, the protein concentration was measured with Pierce Rapid Gold BCA Protein Assay Kit (Thermofisher) and adjusted using Western blot lysis buffer. Supernatants were centrifuged on top of a 20% sucrose layer at 21,000 g for 2 h. The viral pellet was then lysed in Western blot lysis buffer with 4× Protein Sample Loading Buffer (LICOR) supplemented with 10% β-mercaptoethanol (Sigma Aldrich) and heated at 95°C for 5 min.

### CellTiter-Glo luminescent cell viability assay

Cell viability was assessed using CellTiter-Glo Luminescent Cell Viability Assay (Promega G7571), following the manufacturer’s protocol. Briefly, 2 days after IWR-1 treatment, 0.3 million uninfected primary CD4+ T cells in 150 µL culture medium were mixed with 150 µL of CellTiter-Glo Reagent. From this mixture, 50 µL was transferred into fresh plates in triplicate. After a 10-min incubation at room temperature, luminescence was measured using an Orion microplate luminometer.

### Flow cytometry

Approximately 200,000 cells were harvested, washed once with PBS, and stained for 30 min at RT in the dark with eBioscience Fixable viability dye 780 (ThermoFisher Scientific, 65-0865-18) and anti-CD4 antibody (PerCP-Cy5.5, Biolegend #317428). Afterward, cells were washed twice with PBS and permeabilized for 20 min with 200 μL BDCytofix/Cytoperm Fixation/Permeabilization Solution Kit (BD Biosciences, 554714) at RT. Cells were washed twice with 200 μL 1× Perm/Wash solution and stained 1 h at 4°C with anti-capsid antibody (KC57-FITC, Beckman Coulter, 6604667). After washing twice with 1× Perm/Wash solution, cells were resuspended in PBS and measured with BD FACSCanto II Flow Cytometer (BD Biosciences).

### Flow cytometry analysis for CD4 and CCR5 expression

CD4 and CCR5 expression in CEM-M7, SupT-1, THP1, and Jurkat cells was determined using flow cytometry analysis. One million parental cells or cells stably expressing Cas9 were washed with 1 × PBS and stained against CD4, CCR5, and CXCR4 surface proteins for 60 min at 4°C (anti-CD4: BioLegend, Cat# 317428, 1:20 dilution; anti-CCR5: BioLegend, Cat# 313708, 1:5 dilution) or isotype controls (BD Biosciences, Cat# 555058, 1:5 dilution; Invitrogen, Cat# Z25041A, 1:20 dilution). Living cells were determined by live-dead staining using the eBioscience Fixable viability dye 780 (1:1,000) for 30 min at RT in the dark prior to antibody incubation. Right before the measurement, cells were washed twice with 1 × PBS and resuspended in 1 × PBS containing BSA.

### Next generation sequencing

NGS was performed using the Illumina NextSeq2000 platform with 60 base-pair paired-end runs. Raw reads were demultiplexed, trimmed, groomed according to quality, and aligned to the custom library sequences using the MAGeCK algorithm suite on the Galaxy platform. Individual read counts were determined and median-normalized to account for differences in library size and read count distributions. Individual sgRNAs targeting the same gene were aggregated, and a variance model based on a negative binomial distribution was applied to statistically assess differences between control (input) and experimental conditions (different days). Gene targets were ranked by MAGeCK based on their *P*-values via a modified robust ranking aggregation (RRA) algorithm (α-RRA) to identify significantly enriched genes. Overrepresented sgRNA sequences compared to the input control represent viruses that had a sgRNA targeting a gene that restricts viral replication. Volcano plots, correlation analyses, dotplots, and heatmaps were generated using R version 4.4.2, ggplot2 version 3.5.2.

### SDS-PAGE and immunoblotting

Western blotting was performed as previously described (70). In brief, whole cell lysates were mixed with 4× Protein Sample Loading Buffer (LI-COR, at a final dilution of 1×) supplemented with 10% β-mercaptoethanol (Sigma Aldrich), heated at 95°C for 5 min, separated on NuPAGE 4% ± 12% Bis-Tris Gels (Invitrogen) for 90 min at 100 V, and blotted onto Immobilon-FL PVDF membranes (Merck Millipore). The transfer was performed at a constant voltage of 30 V for 30 min using a semi-dry transfer system. For larger proteins (Cas9, EHMT2), transfer was performed at a constant Amperage 0.4 A for 2 h using a wet transfer system. After the transfer, the membrane was blocked in 1% Casein in PBS (Thermo Scientific).

### CRISPR/Cas9 KO in SupT1-CCR5-Cas9

One million SupT1-CCR5 cells stably expressing Cas9 were electroporated with a HiFi Cas9 Nuclease V3 (IDT)/gRNA complex (80 pmol Cas9, 300 pmol gRNA; Lonza), using either a non-targeting or gene-specific sgRNA. Electroporation was performed using the Amaxa 4D-Nucleofector system with the Human Activated T Cell Nucleofector Kit (P3; Lonza, #V4XP-3032), pulse code EO115. Three days post-electroporation, 0.6 million cells per condition were infected with SIVcpz*Ptt* MB897 or HIV-1 NL4-3. Next day, cells were washed three times and either treated with IFN-β at 20 U/mL or left untreated. From 3 to 9 days post-infection, cells were either left untreated or re-treated with IFN-β every 3 days, as indicated. Culture supernatants were collected at 3-day intervals, and infectious virus production was quantified using the TZM-bl reporter cell assay.

### CRISPR/Cas9 KO in primary human CD4+ T cells

CD4^+^ T lymphocytes were isolated from healthy donors. Cells were stimulated for 3 days with IL-2 (10 ng/mL; Miltenyi Biotec, #130-097-745) and anti-CD3/CD28 beads (Gibco, #11132D). Cultures were maintained in RPMI-1640 medium supplemented with 20% FCS and IL-2 (10 ng/mL). One million stimulated primary CD4^+^ T cells were electroporated with a HiFi Cas9 Nuclease V3 (IDT)/gRNA complex (80 pmol Cas9 300 pmol gRNA; Lonza), using either a non-targeting or gene-specific sgRNA. Electroporation was performed using the Amaxa 4D-Nucleofector system with the Human Activated T Cell Nucleofector Kit (P3; Lonza, #V4XP-3032), pulse code EO115. Three days post-electroporation, one million cells per sample were infected with the indicated SIV or HIV-1 strains. From 3 to 12 days post-infection (dpi), supernatants were collected every 3 days and infectious virus production was quantified using the TZM-bl reporter cell assay.

### TV kinetics in primary human CD4+ T cells

CD4+ T lymphocytes were isolated and stimulated as described above. Stimulated primary human CD4+ T cells were infected with lentiviruses carrying Cas9. At 3 days post-infection, 0.5 million cells per sample were infected with the indicated SIV or HIV strains. From 3 to 11 days post-infection (dpi), supernatants were collected every 3 or 2 days, and infectious virus yield was measured on TZM-bl reporter cells.

### Statistics

Statistical analyses were performed using GraphPad PRISM 10 (GraphPad Software). *P*-values were determined using a two-tailed Student’s *t*-test with Welch’s correction or two-way ANOVA with Sidak’s multiple comparisons. Unless otherwise stated, data are shown as the mean of at least three independent experiments ± SEM. Significant differences are indicated as *, *P* < 0.05; **, *P* < 0.01; ***, *P* < 0.001. Statistical parameters are specified in the figure legends.

## Data Availability

All data generated or analyzed in this study are included in the article and its supplemental material. Materials and reagents generated in this study are available from the corresponding author upon reasonable request and in compliance with institutional and regulatory requirements.

## SUPPLEMENTAL MATERIAL

Supplemental figures jvi.00314-26-s0001.pdf Fig. S1 to S10.

Table S1 jvi.00314-26-s0002.xlsx MAGeCK scores.

ASM does not own the copyrights to Supplemental Material that may be linked to, or accessed through, an article. The authors have granted ASM a non-exclusive, world-wide license to publish the Supplemental Material files. Please contact the corresponding author directly for reuse.
