# Supplementary material for: Replication-competent SIVcpz CRISPR screen identifies barriers to successful cross-species transmission
Source: J Virol. 2026 May 7;100(6):e00314-26. doi: 10.1128/jvi.00314-26 (PMC13288819; doi:10.1128/jvi.00314-26)
Supplement: Supplemental figures — Fig. S1 to S10. [file jvi.00314-26-s0001.pdf]

Replication-competent SIVcpz CRISPR screen identifies barriers to successful cross-species transmission

Qinya Xie, Qingxing Wang, Sabrina Noettger, Guillermo Gosálbez, Annika Betzler, Meta Volcic, Dorota Kmiec, Stefan Krebs, Alexander Graf, Dila Gülensoy, Gilbert Weidinger, Konstantin M.J. Sparrer and Frank Kirchhoff

Ten supplemental figures

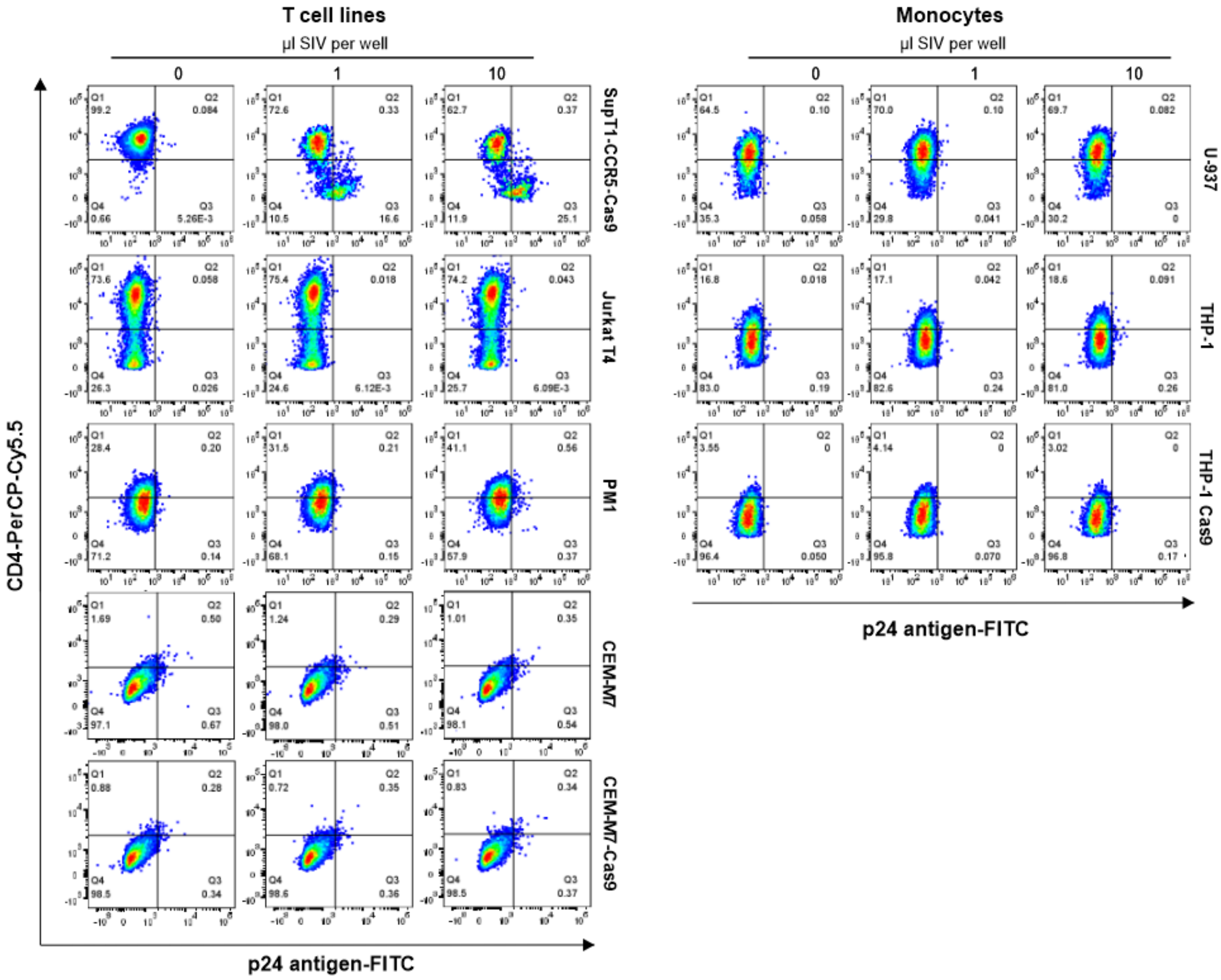

Figure S1. Susceptibility of human cell lines to SIVcpz*Ptt* MB897 infection. Flow cytometry analysis of CD4 and p24 capsid antigen expression levels in the indicated human cell lines after exposure to SIVcpz*Ptt* MB897.

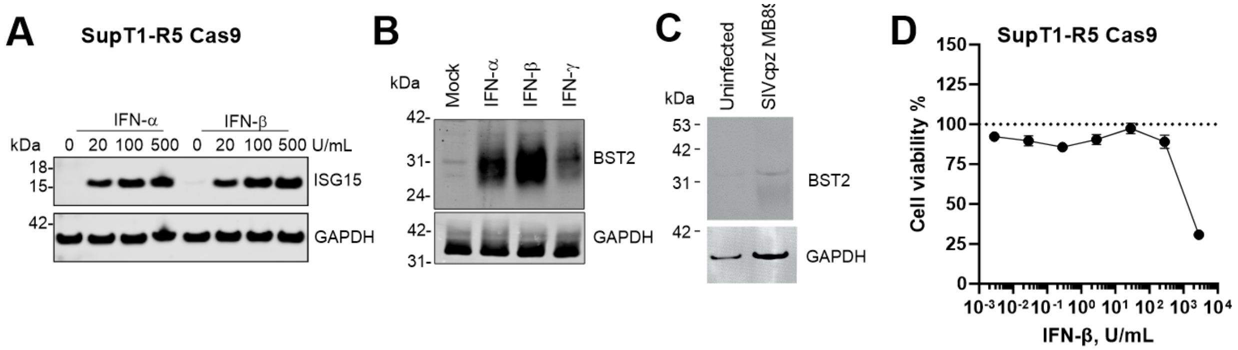

Figure S2. Responsiveness of SupT1-CCR5 Cas9 to type I IFNs. (**A**) Representative Western blot of ISG15 and GAPDH expression levels in SupT1-CCR5-Cas9 cells treated with the indicated doses of type I IFNs. (**B**) One million SupT1-CCR5-Cas9 cells were treated with indicated IFNs at 100U/ml of left untreated for 24h. Whole cell lysates were harvested for western blot. (**C**) Immunoblot from SupT1-CCR5 cells stably expressing Cas9 that were left uninfected or infected with SIVcpz MB897 for 6 days. (**D**) Effect of IFN-ß on the viability of SupT1-CCR5-Cas9 cells at 12 days post treatment. Shown are mean values (±SEM) from triplicate measurements.

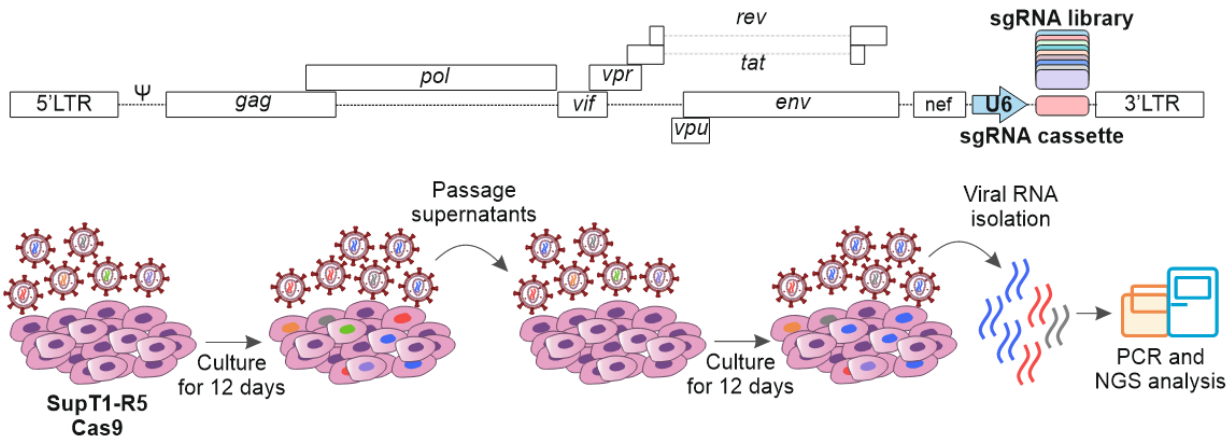

Figure S3. Schematic of proviral SIVcpz*Ptt* MB897 TV constructs and cell culture passaging. Proviral SIVcpz*Ptt* MB897 constructs are engineered to contain the sgRNAs expression cassette between the *nef* gene and the 3`LTR. To produce virus stocks, HEK293T cells are transfected with the viral libraries expressing various sgRNAs. The resulting swarms of MB897-sgRNA viruses are cultured for 12 days in Cas9-expressing cells in the presence or absence of IFN-β. Thereafter, the culture supernatant is used to initiate a 2nd round of culture. Cells and viral supernatants are harvested every three days and the frequencies of sgRNAs are determined by next-generation sequencing. Note that the U6-sgRNA-scaffold region is not to scale.

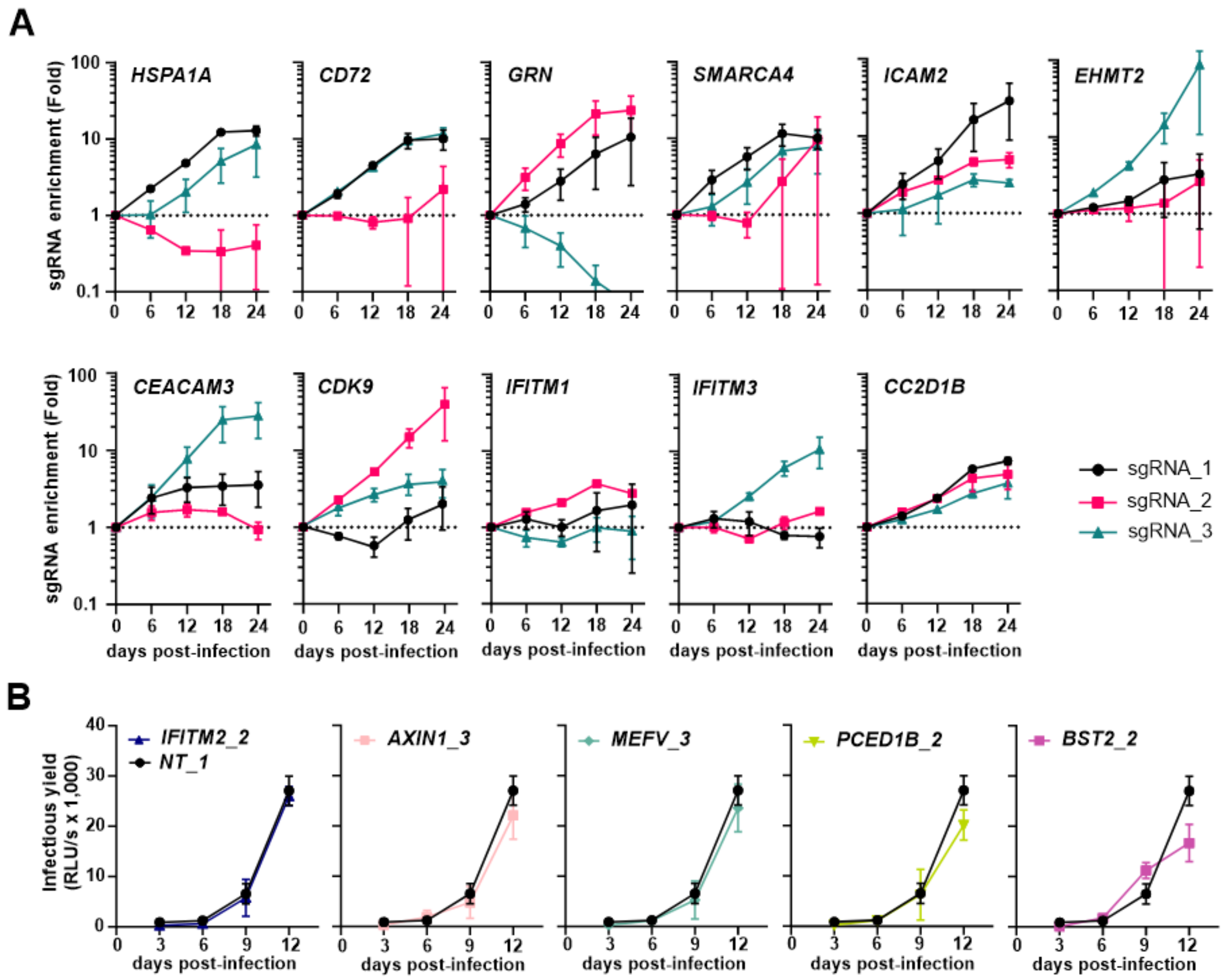

Figure S4. Enrichment of specific sgRNAs and enhancement of viral replication fitness. (**A**) Read-counts relative to input virus from the MAGeCK analysis showing the enrichment of sgRNAs targeting the indicated cellular genes over time. (**B**) SupT1-R5 cells were infected with SIVcpz*Ptt* MB897 constructs expressing the indicated targeting or a non-targeting control sgRNA. Infectious virus yield was measured using TZM-bl repoter cell. Symbols represent the mean of three independent experiments ±SEM.

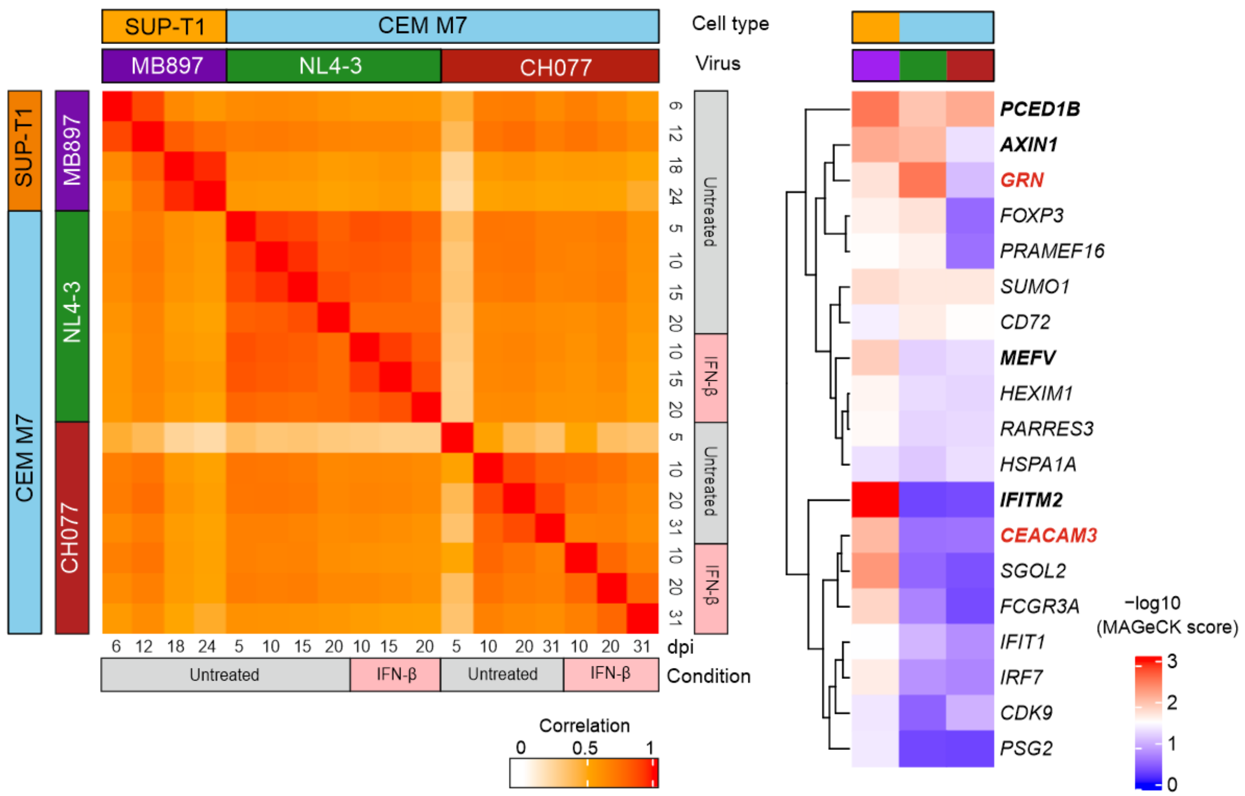

Figure S5. Comparison between SIVcpz*Ptt* MB897 and HIV-1 screens. (Left) Correlation of MAGeCK scores across all genes between the different HIV-1- and SIVcpz-driven screens. (Right) MAGeCK scores of all factors from the SIVcpz screen with log2 (fold-change) > 0 and p-value < 0.05 at 12 days post-infection, displayed across all screens.

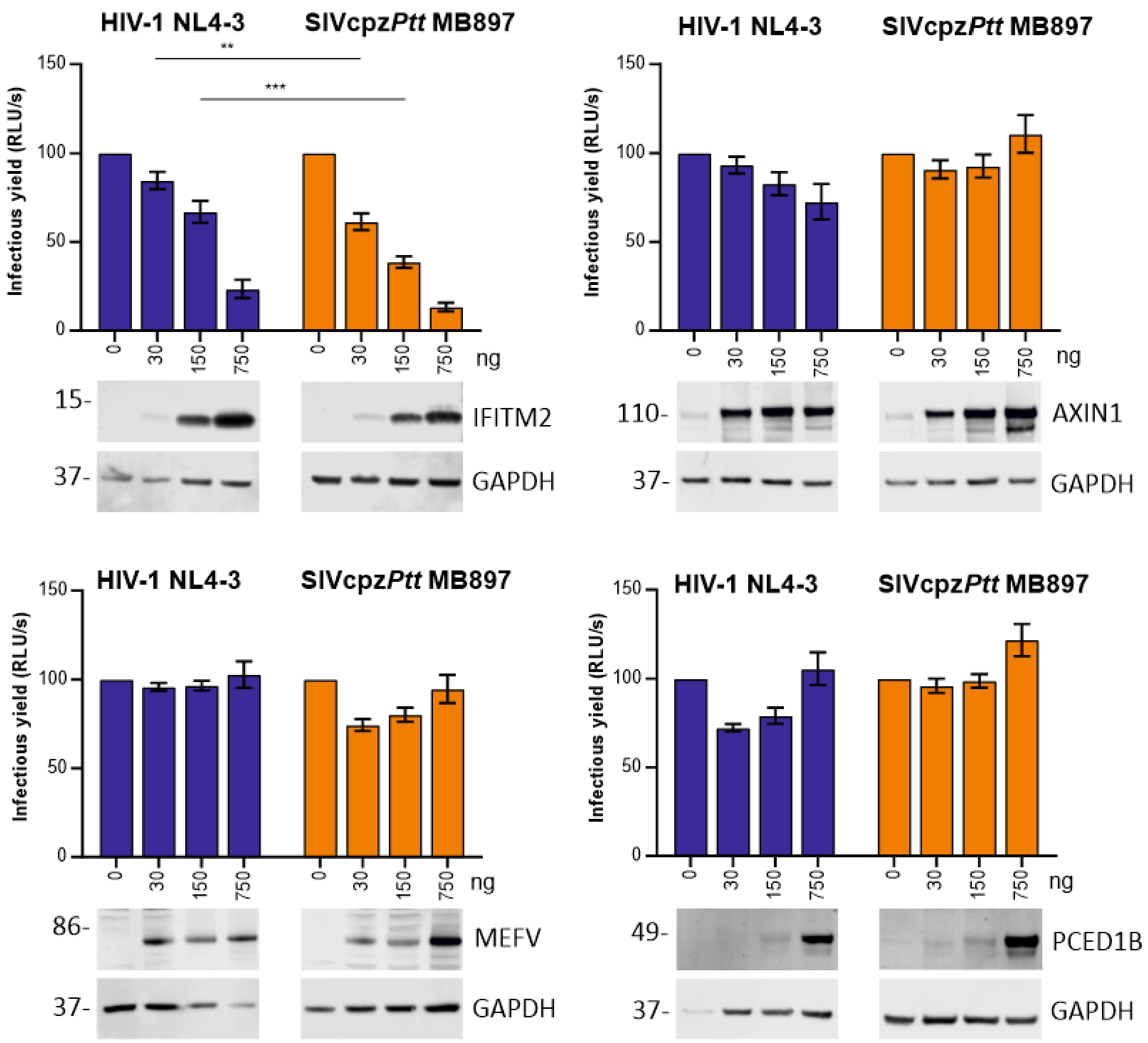

Figure S6. Effects of top hits overexpression in HIV-1 NL4-3 and SIVcpz MB897 production. HEK293T cells were co-transfected with increasing amounts of indicated expression vectors together with HIV-1 NL4-3 or SIVcpz MB897.2 proviral constructs. Supernatants were harvested at 2 days post-transfection, and infectious virus yields were measured on TZM-bl reporter cell. Shown are mean values (±SEM) from four independent experiments.

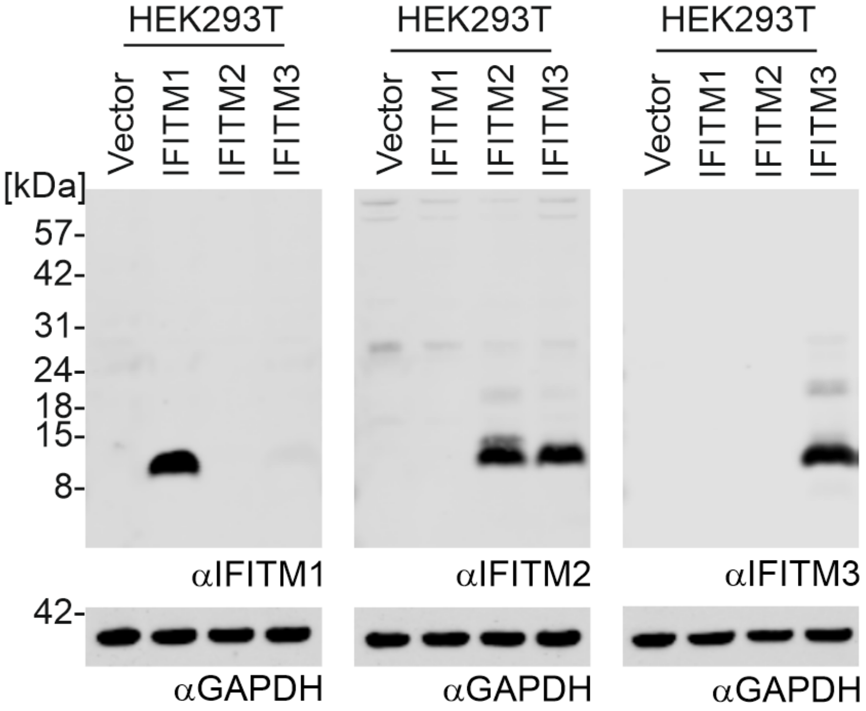

Figure S7. Expression of exogenous IFITMs proteins. HEK293T cells were co-transfected with IFITM expression vectors together with different IMCs. Whole cell lysates were harvested at 2 days post-transfection for western blot.

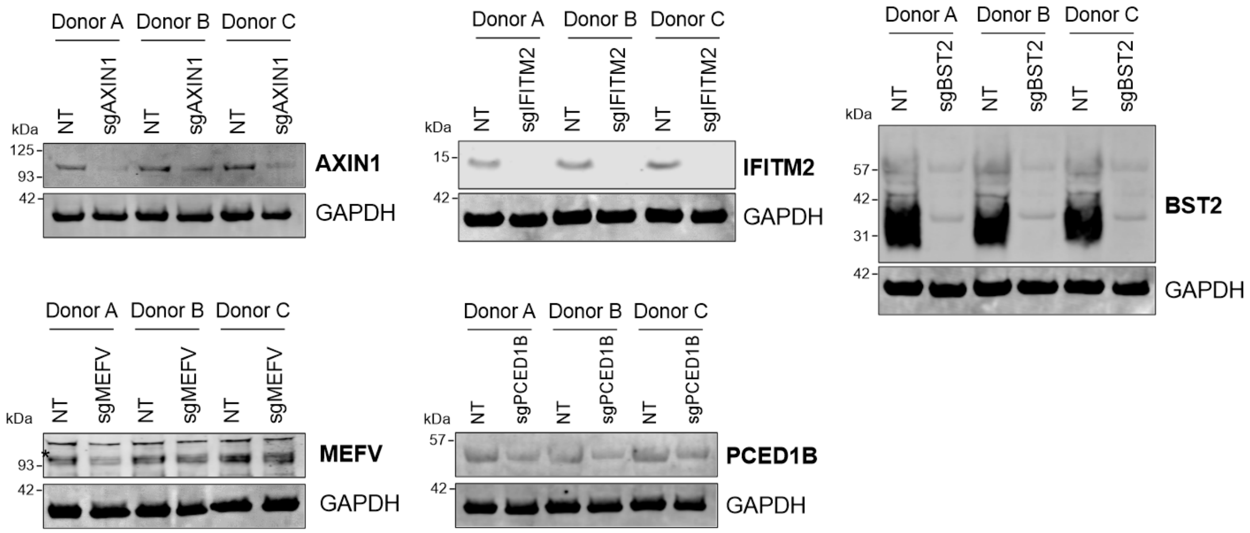

Figure S8. Knocking out efficiency in primary CD4+ T cells. Primary CD4+ T cells were electroporated with sgRNA targeting indicated genes or a non-targeting control. Whole cell lysates were harvested at 3 days post-electroporation for western blot.

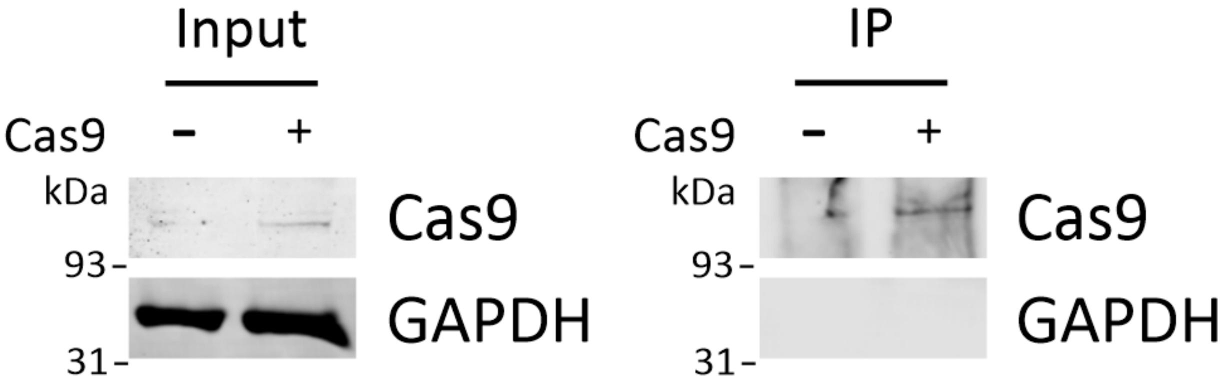

Figure S9. Verification of Cas9 expression in CD4+ T cells. Left: primary CD4+ T cells were transduced by a VSVg-pseudo-typed lentiviral vector expressing Cas9 or left untreated, harvested 2 days later, and analyzed by western blot. Right: to increase Cas9 signal intensity and ensure specificity, 1000 μg of protein lysates were subjected to immunoprecipitation using a Cas9 antibody.

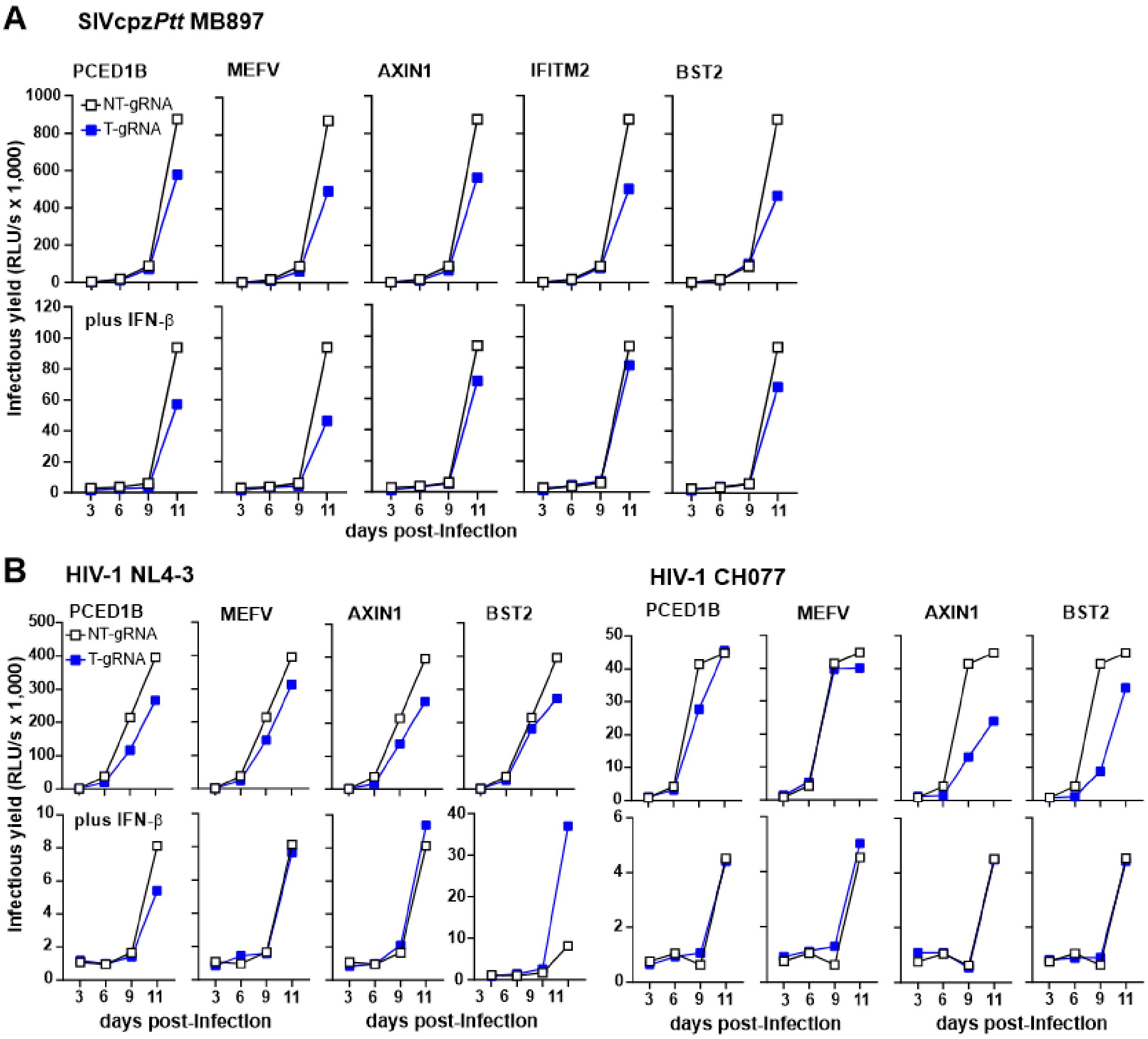

Figure S10. Effect of sgRNAs on SIVcpz and HIV-1 replication in primary human CD4+ T cells in the absence of Cas9. (**A**) Replication of SIVcpz*Ptt* MB897 sgRNA constructs targeting the indicated genes in primary human CD4+ T cells. Infectious virus yield was quantified by TZM-bl assay. (**B**) Replication of HIV-1 sgRNA constructs expressing the same sgRNAs as SIVcpz*Ptt* MB897.
